# Ultrathin and capacity-tunable lithium metal wires for lithium-based fiber batteries

**DOI:** 10.1093/nsr/nwae480

**Published:** 2024-12-31

**Authors:** Chuanfa Li, Qian Ye, Jiaqi Wang, Xinlin Huang, Tianbing Song, Kun Zhang, Pengzhou Li, Yanan Zhang, Xiaocheng Gong, Yi Jiang, Yue Gao, Huisheng Peng, Bingjie Wang

**Affiliations:** State Key Laboratory of Molecular Engineering of Polymers, Department of Macromolecular Science, Institute of Fiber Materials and Devices, and Laboratory of Advanced Materials, Fudan University, Shanghai 200438, China; State Key Laboratory of Molecular Engineering of Polymers, Department of Macromolecular Science, Institute of Fiber Materials and Devices, and Laboratory of Advanced Materials, Fudan University, Shanghai 200438, China; State Key Laboratory of Molecular Engineering of Polymers, Department of Macromolecular Science, Institute of Fiber Materials and Devices, and Laboratory of Advanced Materials, Fudan University, Shanghai 200438, China; State Key Laboratory of Molecular Engineering of Polymers, Department of Macromolecular Science, Institute of Fiber Materials and Devices, and Laboratory of Advanced Materials, Fudan University, Shanghai 200438, China; State Key Laboratory of Molecular Engineering of Polymers, Department of Macromolecular Science, Institute of Fiber Materials and Devices, and Laboratory of Advanced Materials, Fudan University, Shanghai 200438, China; State Key Laboratory of Molecular Engineering of Polymers, Department of Macromolecular Science, Institute of Fiber Materials and Devices, and Laboratory of Advanced Materials, Fudan University, Shanghai 200438, China; State Key Laboratory of Molecular Engineering of Polymers, Department of Macromolecular Science, Institute of Fiber Materials and Devices, and Laboratory of Advanced Materials, Fudan University, Shanghai 200438, China; State Key Laboratory of Molecular Engineering of Polymers, Department of Macromolecular Science, Institute of Fiber Materials and Devices, and Laboratory of Advanced Materials, Fudan University, Shanghai 200438, China; State Key Laboratory of Molecular Engineering of Polymers, Department of Macromolecular Science, Institute of Fiber Materials and Devices, and Laboratory of Advanced Materials, Fudan University, Shanghai 200438, China; State Key Laboratory of Molecular Engineering of Polymers, Department of Macromolecular Science, Institute of Fiber Materials and Devices, and Laboratory of Advanced Materials, Fudan University, Shanghai 200438, China; State Key Laboratory of Molecular Engineering of Polymers, Department of Macromolecular Science, Institute of Fiber Materials and Devices, and Laboratory of Advanced Materials, Fudan University, Shanghai 200438, China; State Key Laboratory of Molecular Engineering of Polymers, Department of Macromolecular Science, Institute of Fiber Materials and Devices, and Laboratory of Advanced Materials, Fudan University, Shanghai 200438, China; State Key Laboratory of Molecular Engineering of Polymers, Department of Macromolecular Science, Institute of Fiber Materials and Devices, and Laboratory of Advanced Materials, Fudan University, Shanghai 200438, China

**Keywords:** ultrathin Li wires, tunable capacities, precise prelithiation, high-energy-density fiber batteries

## Abstract

Ultrathin lithium (Li) metal wires with tunable capacities have great promise for precise prelithiation of fiber anodes and high-energy-density Li-based fiber batteries. However, the application of Li metal in fiber batteries faces great challenges due to its mechanical fragility and the resulting limited micro-dimension manufacturing capability. These challenges impede the production of ultrathin Li wires with adjustable Li contents to match the capacities of Li-based fiber batteries. Herein, silver-plated aramid yarns (Ag/AYs) are employed to load Li metal for producing ultrathin Li wires. The bundled structure of Ag/AYs leads to the adjustable volume of oriented voids within the fibers, thus resulting in accurately tunable capacities (0.0048–2.4 mAh cm^−1^) and diameters (20–534 μm) of Li wires. Such thin Li wires are used to precisely compensate for Li loss during the formation cycle of the fiber graphite anodes, thereby improving the initial Coulombic efficiency from ∼88% to ∼100%. Additionally, when employed as anodes, these Li wires enabled the fiber batteries to exhibit exceptional cycling stability for 150 cycles under a relatively low negative/positive ratio of 2.06, while achieving a high energy density of 139.822 Wh kg^−1^ based on the total mass of the battery.

## Teaser

This work presents a protocol to fabricate ultrathin, capacity-tunable Li metal wires, enabling precise prelithiation of fiber Li-ion batteries and enhancing the energy density of fiber Li-metal batteries.

## INTRODUCTION

Flexible, thin and lightweight fiber Li-ion batteries (FLIBs) have recently emerged as significant candidates for power supply systems in portable and wearable electronics because of their omnidirectional flexibility, high permeability and weavability [1–3]. Despite great achievements, the energy-storing performance of FLIBs still lags behind that of commercial bulk Li-ion batteries, failing to keep pace with the rapidly growing energy demands of wearable electronics [2–11]. However, further improvement in the energy density of FLIBs is hindered by the limited theoretical specific capacity of traditional electrode materials, such as graphite [12–14], as well as the significant capacity loss (∼10%) during the formation cycle of the graphite anode [15–18]. In light of these challenges, there is an urgent need to develop novel high-energy-density anode materials and/or Li-compensating materials well-matched to the 1D configuration of fiber batteries. Li metal wire is the most efficient fiber Li compensating material to offset the initial active Li loss of FLIBs and is a promising fiber anode material for high-energy-density fiber Li metal batteries (FLMBs) [15–23].

The energy density of Li-based fiber batteries, including FLMBs and FLIBs, is significantly impacted by the active Li content. Excessive Li content of the fiber Li metal anode in FLMBs due to the use of thick Li rods, or insufficient Li content in the FLIBs stemming from the loss of active Li during the formation cycle, both greatly compromise the overall energy density of batteries. Specifically, when serving as anodes, Li metal fiber must possess a low Li content to achieve a low negative/positive (N/P) ratio (≤2) [13,24], thus unlocking the potential of Li anodes for considerably enhancing the energy density of FLMBs [12,25]. As a Li-compensating material, the Li metal fiber should contain a lower and suitable amount of Li to precisely compensate for the capacity loss of FLIBs during the formation cycle (∼10% of the capacity of the whole battery). Therefore, the fabrication of Li wires with adjustable Li contents, namely, tunable capacities (within 10% to 200% of the capacities of the batteries), is of great importance for enhancing the energy density of fiber batteries. However, because of its mechanical fragility and sticky nature stemming from the high homologous temperature at room temperature, Li metal generally exhibits limited micro-dimension manufacturing capability.

The existing Li metal fibers show diameters as large as 3 mm, delivering an extremely high linear capacity of 45 mAh cm^−1^. This excessive capacity of the Li metal rod far exceeds that of the fiber cathode, thus leading to an excessively high N/P ratio and significantly reduced energy density of FLMBs. Moreover, it is more challenging to obtain thinner Li metal fibers with lower capacities to accurately compensate for the loss of active Li in FLIBs during the formation cycle. Excessive Li compensation in FLIBs may lead to Li deposition on the graphite anode, shortening battery life and even posing risks of short circuits. Despite some studies reporting the fabrication of Li metal fibers with low diameters (∼300 μm) and capacities (∼1 mAh cm^−1^) [17,18], neither the diameter nor the capacity of these Li wires can be tuned to meet the demands of high-energy-density FLMBs and the precise prelithiation of FLIBs. Therefore, it remains a great challenge to produce ultrathin Li metal wires with tunable diameters and capacities to match the requirements of Li-based fiber batteries.

**Figure 1.**
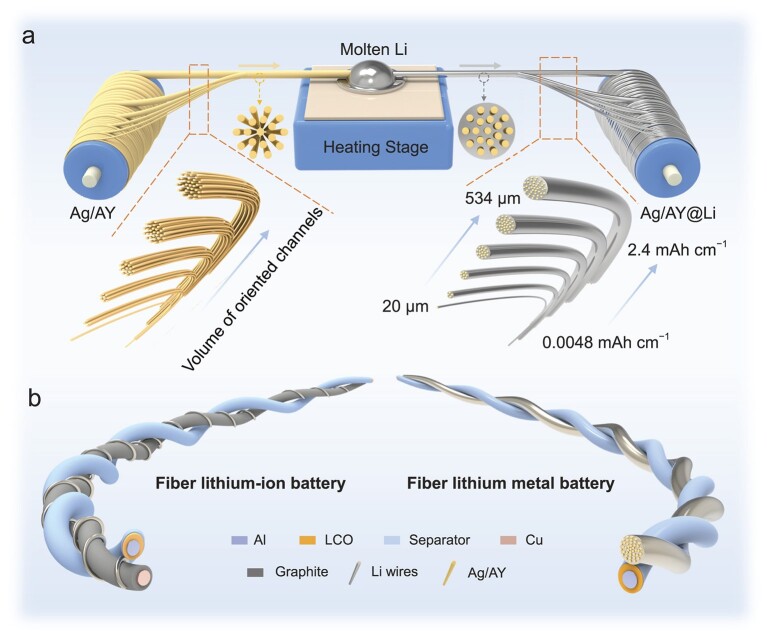
(a) Schematic showing the fabrication process of ultrathin Li wires with tunable diameters and capacities. (b) Li wires work as fiber Li compensating materials and fiber anodes to be applied in FLIBs and FLMBs.

Herein, commercially available silver-plated aramid yarns (Ag/AYs) are introduced as loading scaffolds of Li metal to produce Li wires through a molten-infusion process (Fig. 1a). Benefiting from the bundled structure of fibers, the volume of oriented channels within bundled Ag/AYs can be adjusted to precisely tune capacities and diameters of the Li wires. As a result, Li wires with tunable capacities ranging from 0.0048 to 2.4 mAh cm^−1^ have been achieved, corresponding to ultrathin and controllable diameters ranging from 20 to 534 μm, which overcomes the intrinsic micro-dimension manufacturing limitations of Li metal. Moreover, our Li wires also exhibit a high mechanical strength of 217 MPa, which is two orders of magnitude higher than that of the commercial Li rod (6.03 MPa), accompanied by outstanding flexibility. More importantly, the Li wire can be produced continuously, highlighting their potential for scalable manufacturing in the fiber battery industry. The controllable and ultralow capacities of these Li metal wires make them ideal compensating materials for the initial loss of active Li in the FLIBs (Fig. 1b). With this Li metal wire, the initial 11.66% capacity loss could be recovered for the fiber graphite anodes. Additionally, when employed as anodes in FLMBs (Fig. 1b), these ultrathin Li wires enable the batteries to exhibit not only a high cycling stability with a capacity retention of 70.3% after 150 cycles under a relatively low N/P ratio of 2.06, but also an ultrahigh energy density of 139.822 Wh kg^−1^ based on the total mass of the battery.

## RESULTS AND DISCUSSION

### Fabrication of ultrathin Li wires with tunable diameters and capacities

The fabrication process of ultrathin Ag/AY@Li wires with controllable capacities and diameters is illustrated in Fig. 1a. We selected AY as the matrix to load molten Li due to its exceptional heat resistance, chemical stability and mechanical strength [26,27]. Electrochemical stability is also a crucial consideration for the use of AY in chemical batteries. Cyclic voltammetry was performed and the AY displayed a wide electrochemical stability window ranging from a Li plating potential of −0.5 V (vs. Li^+^/Li) to an electrolyte decomposition potential of 4.6 V (vs. Li^+^/Li) (Fig. S1), highlighting the high electrochemical stability of AY. However, the bare AY matrix cannot be wetted by molten Li due to the high surface tension of molten Li on AY, or the lithiophobic nature of AY (Fig. S2). Therefore, the lithiophobic surface of AY needed to be modified to provide a large driving force for Li infusion into the fiber matrix. Previous studies have shown that the employment of an Ag layer on a lithiophobic surface can significantly improve the wettability of matrix with molten Li and thus enable successful Li infusion [28]. Consequently, Ag/AYs with an Ag plating layer are employed as the host to load molten Li.

Thermogravimetric analysis (Fig. S3) confirms the thermal stability of Ag/AYs up to 525°C, well above the melting point of Li (180°C) and the operating temperature (200–300°C) during the molten-infusion process. The high thermal stability ensures that the Ag/AY fiber matrices can withstand the high temperature of molten Li. The morphology of the Ag/AY matrix was characterized by scanning electron microscopy (SEM). Evident core-sheath structure is observed from the cross-sectional SEM images (Fig. S4), where the AY cores appear darker in color, and the Ag sheaths are brighter. The average diameter of the filament in the Ag/AY matrix was measured as 18.8 μm (Fig. S5b and d). By comparing this with the diameter of the pristine aramid filament (14.2 μm) (Fig. S5a and c), the thickness of the Ag plating layer was estimated to be 2.3 μm. Further structural analysis was performed using X-ray diffraction (Fig. S6). The diffraction peaks observed at 2θ values of 38.11°, 44.30°, 64.44°, 77.40° and 81.54° correspond to the (111), (200), (220), (311) and (222) planes of Ag, consistent with the standard PDF card (ICDD PDF# 99–0094). Additionally, peaks at 43.32°, 50.45°, 74.12° and 89.94° are attributed to the characteristic diffraction peaks of Cu (ICDD PDF# 99–0034). This Cu layer is introduced as a pre-coating to improve the adhesion of the Ag layer to the AY surface. The disappearance of the C signal in the energy-dispersive X-ray spectroscopy element mapping for the Ag/AY matrix demonstrates the complete covering of the Ag plating layer (Fig. S5a and b). Benefiting from the uniform and conformal Ag coating layer on each filament of the AY, the Ag/AY matrix exhibits significantly enhanced wettability with molten Li (Fig. S2). In addition, numerous voids can be observed within the Ag/AY matrix, which can be ascribed to the oriented channels between interstacked aramid filaments (Fig. S5b). These well-developed voids provide sufficient capillarity for molten Li infusion and enough space for Li loading. Additionally, the highly oriented channels in the Ag/AY matrix lead to relatively low tortuosity, which significantly facilitates the rapid infusion of Li into the voids of the fiber matrix and boosts the productivity of Li wire. Besides, the Ag/AY matrix demonstrated excellent electrical performance, with its resistance remaining stable at 8 Ω/m for >20 h (Fig. S7). This stability ensures consistent and reliable electronic transport for active Li.

The molten-infusion process is operated by immersing Ag/AYs into molten Li (Fig. 1a). During this process, the molten Li initially reacts with the Ag plating layer and is loaded onto the surface of each aramid filament (Fig. S8a and c). Subsequently, due to the siphonic effect, molten Li is automatically infused into the highly oriented voids of the lithiated Ag/AY matrices and spreads onto the entire frameworks, resulting in the fabrication of Ag/AY@Li wires (Fig. S8a and d). As shown in Fig. S9, the Fourier-transform infrared spectra of the AY obtained from the Ag/AY matrix and the Ag/AY@Li wire are nearly the same, thus further confirming the high thermal and chemical stability of the AY after the molten-infusion process.

**Figure 2.**
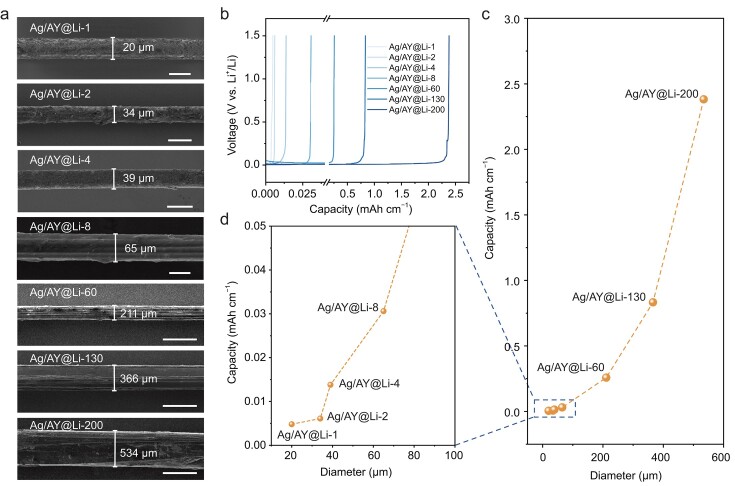
The tunable diameters and linear capacities of Ag/AY@Li wires. (a) SEM images of the Ag/AY@Li wires with different diameters. Scale bars: 20 μm for Ag/AY@Li-1; 50 μm for Ag/AY@Li-2, Ag/AY@Li-4 and Ag/AY@Li-8; 500 μm for Ag/AY@Li-60, Ag/AY@Li-130 and Ag/AY@Li-200. (b) Full Li stripping curves of ultrathin Ag/AY@Li wires with different diameters, showing their tunable linear capacities. (c) Capacities of Ag/AY@Li wires with various diameters and (d) the corresponding local magnification.

In comparison with the commercialized thick Li metal rods with a diameter of ∼3 mm (Fig. S10), our Ag/AY@Li wires display significantly smaller and finely tunable diameters ranging from 20 to 534 μm (Fig. 2a). This precise tunability can be attributed to the controllable void volume of the oriented channels within the Ag/AYs stemming from the bundled structure of the aramid filaments (Fig. S11). Such small diameters of the fabricated Li wires break the inherent producing limitations of Li metal and underscore their potential as micrometer-scale electrodes for fiber battery manufacturing. These micrometer-level Li wires, with diameters of 20, 34, 39, 65, 211, 366 and 534 μm, designated as Ag/AY@Li-1, 2, 4, 8, 60, 130 and 200, respectively, according to the number of the filaments within the Ag/AY matrices, offer a broad range of capacities of 0.0048, 0.0061, 0.012, 0.042, 0.27, 0.822 and 2.4 mAh cm^−1^, respectively (Fig. 2a–d). Furthermore, it is possible to more finely regulate the capacity of Li wires by using the bundled Ag/AYs with smaller incremental changes in the number of filaments (Fig. S12).

This fabrication procedure demands only an argon atmosphere. And the Ag/AYs are commercially available. These advantages greatly enhance its cost-effectiveness and reliable scalability. As such, we demonstrated the continuous preparation of a coil of an Ag/AY@Li wire with a length exceeding 5 m within 20 min (Fig. S13), highlighting our molten-infusion process as a promising strategy for manufacturing micrometer-scale Li metal electrodes for the fiber battery industry.

In addition to their finely controllable diameters and capacities, the obtained Ag/AY@Li wires also possess remarkable mechanical properties derived from the mechanically robust AY substrates. As shown in Fig. S14, the stable structure of the Li wires remains intact even after undergoing bending and twisting, underscoring the outstanding flexibility of the as-fabricated Ag/AY@Li wires. Furthermore, after being repeatedly bent 50 times, no cracks are observed in the Ag/AY@Li wire, further demonstrating its superior mechanical flexibility (Fig. S15). In stark contrast, fracture occurs in the commercially available Li rods after repeated bending. Additionally, the Ag/AY@Li wires exhibit an impressively high tensile strength of 217 MPa, which is two orders of magnitude higher than that of commercial Li rods (6.03 MPa) (Fig. S16). Taken together, these results indicate that the Ag/AY matrices provide superior durability and recoverability compared with Li rods, which is crucial for their application in producing fiber batteries.

### Ultrathin Ag/AY@Li wire enables precise prelithiation of fiber graphite anodes

**Figure 3.**
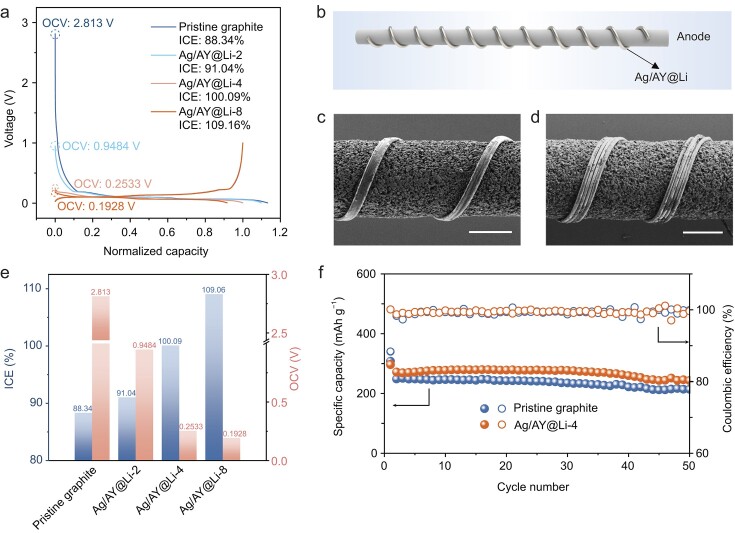
Ultrathin Ag/AY@Li wire enables precise prelithiation and an ideal ICE for fiber graphite anodes. (a) Voltage profiles of fiber graphite anodes during the first cycle, using Ag/AY@Li wires with different diameters for prelithiation. The decreased OCVs (indicated by dashed circles) are due to the reduced electrochemical potentials of lithiated graphite. (b–d) Schematic illustrating prelithiation of a fiber graphite anode with an Ag/AY@Li wire (b) and the corresponding SEM images before (c) and after (d) the Li compensating process. Scale bar: 200 μm. (e) Comparisons of the ICE and OCV of graphite||Li half-cells using Ag/AY@Li wires with different diameters for anode prelithiation. (f) Galvanostatic cycling of fiber graphite electrodes with and without prelithiation.

Benefiting from their tunable diameters and ultralow capacities, these Ag/AY@Li wires are well-suited for prelithiating the anodes of FLIBs, enabling precise compensation for the capacity loss in FLIBs due to the solid electrolyte interface (SEI) formation during the first cycle. The graphite || Li half-cell exhibits a relatively low initial Coulombic efficiency (ICE) of 88.34% without prelithiation, indicating that 11.66% of the active Li is consumed during the formation cycle (Fig. 3a). This loss corresponds to a deviation of ∼0.040 mAh cm^−1^ between the charge and discharge capacity of the first cycle in a high-loading fiber graphite anode (1.0 mg cm^−1^), which has achieved scalable production in our previous work [2].

As shown in Fig. 3b and c, this initial Li loss can be effectively compensated by simply wrapping an Ag/AY@Li wire around the graphite anode. After electrolyte injection, electrons transfer directly from the Ag/AY@Li wire to the graphite anode through the contact points, while active Li^+^ diffuse through the electrolyte from the Ag/AY@Li wire to the graphite anode, contributing to the SEI formation on the anode. This process is thermodynamically spontaneous, driven by the chemical potential difference between the Li wire and the graphite anode (Fig. S17). No Li but the Ag/AY scaffold is observed after overnight electrolyte immersion, indicating complete Li stripping from the Ag/AY@Li wire (Fig. 3d).

By using the Ag/AY@Li-4 wire with a diameter of 39 μm (containing a linear capacity of 0.012 mAh cm^−1^) and a length three times longer than the fiber graphite anode, the half-cell exhibits a significant decrease in open-circuit voltage (OCV) to 0.2533 from 2.813 V (Fig. 3a and e). Additionally, the ICE of the prelithiated anode improves to 100.09%, suggesting full compensation of the initial Li loss. A longer length of the Li wire than that of the fiber graphite anode is necessary for firm wrapping of Li wire around the fiber anode with more contact area, which is important for facilitating homogeneous prelithiation of the anode. To assess the uniformity of prelithiation, SEM imaging and X-ray spectroscopy element mapping were performed on three distinct regions of the prelithiated fiber anode after unwrapping the Li wire (Fig. S18a). The SEM images display consistent microtopographies across these regions, and the element mapping confirms a uniform distribution of C, O and F elements. These findings underscore the effectiveness of our method in achieving uniform prelithiation (Fig. S18b). Importantly, precisely prelithiated graphite anodes exhibit no obvious compromise in anode cycling stability (Fig. 3f). And the impact of the Ag/AY wire on cycling capacity remains negligible over extended cycles (Fig. S19).

An Ag/AY@Li-8 wire with a diameter of 65 μm (containing a linear capacity of 0.042 mAh cm^−1^) raises the ICE to 109.16%, suggesting excessive Li compensation, which could lead to unsafe metallic Li deposition in the graphite anode (Fig. 3a and e). Conversely, using an Ag/AY@Li-2 wire with a diameter of 34 μm (containing a linear capacity of 0.0061 mAh cm^−1^), the ICE is only compensated to 91.04%, demonstrating inadequate prelithiation, which could compromise the energy density of the battery (Fig. 3a and e). Generally, precise prelithiation is of great importance to improve the energy density of the battery without compromising its safety and lifespan. Theoretically, this prelithiation approach is estimated to increase energy density by ∼9% for traditional LCO||Gr batteries (Fig. S20). Furthermore, when applied to high-specific-capacity anode materials like silicon-based anodes, this approach enables FLIBs to achieve even higher energy densities.

### Ultrathin Ag/AY@Li wires as anodes enable high-energy-density FLMBs

**Figure 4.**
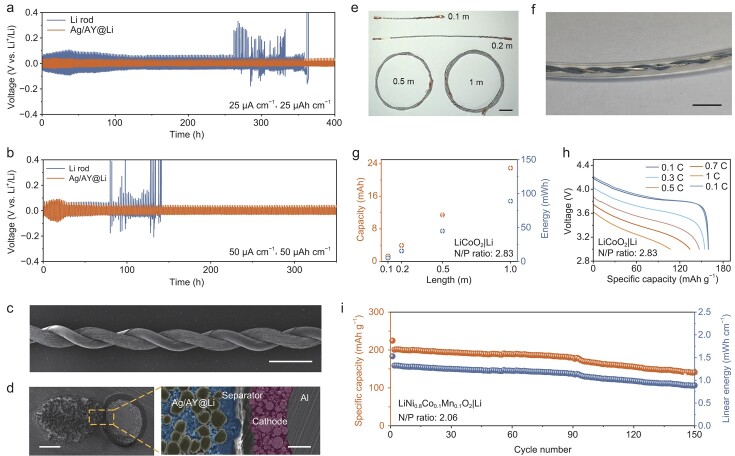
Ultrathin Ag/AY@Li wires as anodes enable high-energy-density FLMBs. (a, b) Voltage–time profiles in symmetric cells with Ag/AY@Li wires and Li rods at various current densities and cycling capacities: (a) 25 μA cm^−1^, 25 μAh cm^−1^; (b) 50 μA cm^−1^, 50 μAh cm^−1^. (c) SEM image of a nude fiber cell. (d) Cross-sectional SEM image of an FLMB. Scale bar, 200 μm. Magnified area (left to right): Ag/AY@Li anode (blue area: Li), separator, cathode (red) and aluminum current collector. Scale bar, 50 μm. (e) Optical image of FLMBs with various lengths. Scale bar, 2 cm. (f) Magnified photograph of an FLMB with a length of 1 m. Scale bar, 4 cm. (g) Capacity and energy of the FLMBs with different lengths. (h) Galvanostatic discharge profiles of FLMBs at increasing discharge rates. (i) Galvanostatic cycling of an FLMB at charging/discharging C rates of 0.3 C/0.5 C.

Furthermore, the micrometer-level diameter, tunable linear capacity, remarkable mechanical properties and higher specific capacity compared with the established graphite make the Ag/AY@Li wire a strong candidate as a fiber Li metal anode for high-energy-density FLMBs (Supplementary Note 1). In LMBs, Li metal anodes often face challenges associated with repeated Li stripping and plating, which results in dendritic Li growth, active Li consumption and eventual battery failure. Therefore, the plating/stripping stability of the obtained Ag/AY@Li wire needs thorough investigation. Typically, the cycling stability of the Ag/AY@Li anode can be evaluated by fiber symmetric cells. When cycled at a current density of 25 μA cm^−1^ and a linear capacity of 25 μAh cm^−1^, the symmetric cell with the Ag/AY@Li wire exhibits an extended lifespan of 400 h with a stable overpotential (∼30 mV) and negligible voltage fluctuation (Fig. 4a). By comparison, the control cell with Li rod electrodes displays a much higher voltage hysteresis accompanied by a sharp increase after ∼260 h, which can be attributed to the gradually increased interfacial resistance caused by the continuous growth/corrosion of Li dendrites and the repeated breakdown/repair of the SEI layer. Even under a markedly increased plating/stripping capacity of 50 μAh cm^−1^, the symmetric cell with Ag/AY@Li wires still delivers superior cycling stability for 350 h with a stable overpotential (∼40 mV) at an increased current density of 50 μA cm^−1^, significantly outperforming the cell with Li rods, which demonstrates a stable cycling life for only ∼80 h and a significantly higher overpotential (Fig. 4b). Remarkably, stable Li plating/stripping for 120 h is also achieved for the cell with the Ag/AY@Li electrodes under a higher current density of 100 μA cm^−1^ with a further increased capacity of 300 μAh cm^−1^ (Fig. S21). The excellent cycling performance strongly clarifies the advantage of the Ag/AY scaffold for stabilizing the Li electrode during the repeated plating and stripping processes. In addition, the fiber symmetric cell with the Ag/AY@Li electrodes exhibits steady and low Li plating/stripping overpotentials of 30, 40, 90 and 140 mV at various current densities of 25, 50, 100 and 150 μA cm^−1^, respectively, with a fixed cycling capacity of 100 μAh cm^−1^ (Fig. S22), verifying fast Li-ion migration kinetics and superior interface properties enabled by the lithiophilic Ag layer derived from the Ag/AY matrix.

To further understand the interfacial properties of the Ag/AY@Li anodes, electrochemical impedance spectroscopy analysis was conducted on the symmetric cell at different cycles (Fig. S23). The cell with the Ag/AY@Li wire demonstrates an interfacial charge transfer resistance of 81 Ω before cycling, significantly lower than that of the cell with Li rod electrodes (175 Ω), indicating the enhanced interfacial Li-ion migration kinetics in the Ag/AY@Li anode. When the symmetric cell with Li rod electrodes is cycled at 50 μA cm^−1^ and 50 μAh cm^−1^, the overall interfacial resistances significantly drop to 154 Ω after the first cycle and further to 93 Ω after 20 cycles, but then increase to 110 Ω after 50 cycles (Fig. S23a). The increased interfacial resistance can be ascribed to the accumulation of ‘dead Li’ on the surface of the Li rod electrode after cycling. This result is consistent with the plating/stripping stability test, in which the overpotential of the symmetric cells with Li rods significantly increases after ∼40 cycles (∼80 h) (Fig. 4b). By contrast, Ag/AY@Li electrodes exhibit significantly lower and more stable interfacial resistances (Fig. S23b), verifying the enhanced stability and migration kinetics of the electrode interface, which can be ascribed to the employment of the Ag/AY host and the derived lithiophilic Ag layer.

The nanomorphologies of the fiber Li metal anodes after cycling were investigated to further demonstrate the superiority of the Ag/AY scaffold in enhancing the stability of Li metal wire anodes. As shown in Fig. S24a and d, both the Li rod and the Ag/AY@Li wire before cycling show smooth surfaces. After 20 cycles at 50 μA cm^−1^ and 50 μAh cm^−1^, the surface of the Li rod electrode evolves from the initial smooth structure to a loosely stacked morphology because of the uneven Li plating and stripping processes (Fig. S24b); this highly porous structure becomes more severe after 50 cycles (Fig. S24c), reflecting the further growth of loosely stacked dendritic Li. The accumulated ‘dead Li’ hinders the transportation of Li ions on the surfaces of Li electrodes, causing high interfacial resistance and increased polarization of the cells, as proven by the electrochemical impedance spectroscopy analyses and the cycling tests (Fig. 4b and Fig. S23a). In sharp contrast, for the Ag/AY@Li electrode, the dendrite-free Li metal is densely plated on the electrode surface after 20 cycles (Fig. S24e). Furthermore, after 50 cycles, a relatively uniform and compact deposition of metallic Li is still retained with a dendrite-free flat surface (Fig. S24f), highlighting the stable Li deposition processes facilitated by the Ag/AY scaffold.

In brief, the improved stripping/plating stability of Ag/AY@Li anodes can be ascribed to the use of Ag/AY as scaffolds. This scaffold not only provides sufficient voids to accommodate the significant volume fluctuations during Li stripping/plating processes, but also derives a lithiophilic Ag layer to stabilize the SEI of the Ag/AY@Li anode.

Benefiting from their remarkable electrochemical stability, the obtained Ag/AY@Li wires have great potential to serve as anodes for high-energy-density FLMBs. Therefore, the Ag/AY@Li-130 wires, with a diameter of 366 μm and a linear capacity of 0.822 mAh cm^−1^, are adopted as the anodes. These fiber anodes are paired with the high-loading (1.61 mg cm^−1^, corresponding to a linear capacity of 0.290 mAh cm^−1^) LiCoO_2_ fiber cathodes, which have achieved scalable production in our previous work [2], to assemble the FLMBs with any length (Fig. 4c–f). In line with our earlier findings in FLIBs, increasing the length of the FLMBs reduces internal resistance (Fig. S25). Notably, these as-fabricated FLMBs have a diameter of only 2 mm, even smaller than that of commercial Li rods, making them suitable for integration into energy-storage fabrics (Fig. 4f). The capacity and energy of the fabricated FLMBs exhibit a linear increase with battery length (Fig. 4g and Fig. S26). FLMBs with lengths of 0.1, 0.2 and 0.5 m display capacities of 1.33, 3.94 and 11.42 mAh, respectively, corresponding to energy outputs of 5.16, 15.48 and 44.86 mWh, respectively. Remarkably, a 1-m-long battery delivers a capacity of 22.94 mAh and an energy output of 88.93 mWh. This energy output is sufficient to power various commercial wearable devices, such as heart rate monitors and muscle oxygen content monitors, for >2 days. In addition, these FLMBs possess remarkable rate performances at continuously varied current rates (Fig. 4h). High capacities of 159.7, 154.5, 147.6, 134.8 and 107.45 mAh g^−1^ are delivered for a 0.5-m-long cell at varied current rates of 0.1, 0.3, 0.5, 0.7 and 1 C (1 C = 180 mAh g^−1^), respectively. After switching back to a low current rate of 0.1 C, a reversible capacity of 159.4 mAh g^−1^ is highly resumed.

Furthermore, LiNi_0.8_Co_0.1_Mn_0.1_O_2_ cathode material, which offers a higher specific capacity, is also used to produce fiber cathodes with a higher loading of 1.77 mg cm^−1^, delivering a significantly increased linear capacity of 0.399 mAh cm^−1^. These high-capacity fiber cathodes are also paired with Ag/AY@Li-130 anodes to assemble FLMBs. The application of these ultrathin Ag/AY@Li-130 wires substantially reduces the N/P ratio to 2.06, a relatively low value, even compared with extensively reported pouch LMBs [29–32]. Such a low N/P ratio unlocks the potential of Li metal anodes and extensively improves the energy density of the FLMBs. Despite the low N/P ratio, FLMBs exhibit remarkable cycling stability, with a capacity retention of 70.3% (calculated based on the second cycle) after 150 cycles (Fig. 4i and Fig. S27), which is comparable with recently reported pouch LMBs [29,33–35]. Notably, such high cycling stability is achieved without the need for external pressure. In sharp contrast, the pouch LMBs generally need high external pressure for optimal cycling performance, which requires additional tools and thus has poor feasibility in practical applications. Additionally, the FLMB demonstrates low and steady polarization throughout the cycles, indicating the highly stable interface of the Ag/AY@Li anodes (Fig. S28). The steady capacity and polarization contribute to stable energy output during cycling (Fig. 4i). Moreover, the as-fabricated FLMB shows an energy density of 139.822 Wh kg^−1^ based on the total mass of the battery, including electrodes, electrolyte, separator, tabs and packaging (Fig. S29 and Table S1). Such a high energy density, to the best of our knowledge, surpasses that of previously reported fiber batteries [2,4,6,7,36–44] and almost approaches the values of commercial bulk Li-ion batteries (150 Wh kg^−1^) (Fig. S30).

In addition to their remarkable electrochemical performance, our FLMBs also possess exceptional flexibility comparable with that of FLIBs [2]. As shown in Fig. S31, the FLMBs demonstrate a capacity retention of 90% even after 100 000 bending cycles at a curvature radius of 1 cm, with steady charging and discharging profiles. Moreover, the FLMBs reliably power a hygrothermograph under various bending angles (Fig. S32), further demonstrating their high flexibility and durability.

### Applications of battery textiles made from FLMBs

**Figure 5.**
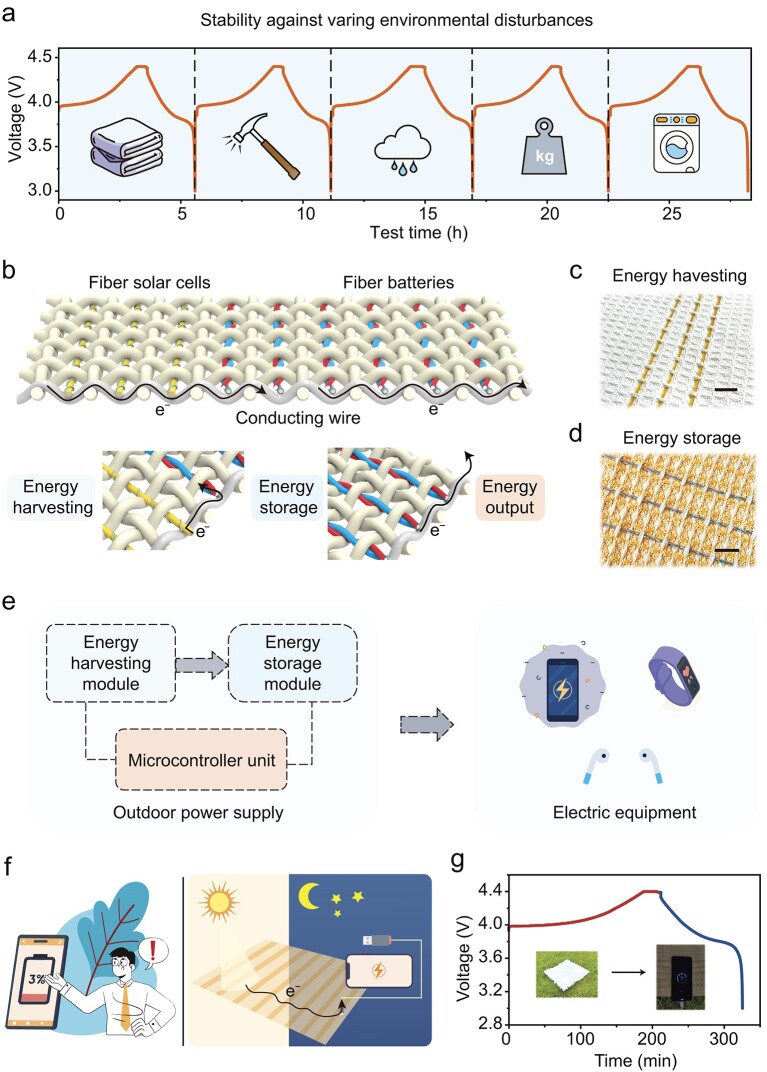
Applications of battery textiles made from FLMBs. (a) The voltage–time profiles of the FLMB-based battery textile show resilience, remaining stable despite exposure to folding, hammering, rain, pressure and washing. (b) The schematic illustrating the operating mechanism of an integrated textile system in the form of a mat consisting of solar cell textiles and battery textiles. Electron flow (arrows) occurs through conducting wires that connect the different textile modules. Photographs of (c) energy-harvesting (solar cell textile) and (d) energy-storage (battery textile) modules of the all-textile integrated system. (e) A circuit diagram of the integrated textile system in (d) and (f) to charge electronic devices like phones, smartwatches and earphones. (f) The schematic demonstrates that the integrated textile works as a self-powered system to solve energy anxiety in outdoor settings, especially at night when solar power is not available. (g) The graph shows the charging curve (red line) of the solar cell textiles and the discharging curve (blue line) of the battery textiles. Inset photos illustrate how solar cell textiles on the upper side of the mat harvest solar energy during the day and store the harvested energy in the FLMB textile on the underside of the mat. The charged mat powers electronic devices at night. Scale bars, 2 cm in (c) and (d).

Because of this outstanding flexibility, we produced a battery textile composed of FLMBs using a commercial weaving machine. This textile demonstrates exceptional stability against various environmental disturbances, such as folding, hammering, rain, pressure and washing (Fig. 5a). Additionally, the battery performance shows no significant degradation after repeated mechanical abrasion, confirming its stability to withstand mechanical wear from regular use (Fig. S33). Given the flexibility and durability of these battery textiles, we envisioned their application in an integrated textile capable of harvesting, storing and discharging energy (Fig. 5b–f). As a proof of concept, we fabricated a picnic mat with an upper layer of solar cell textiles and an under layer of battery textiles (Fig. 5f and g). The mat is designed to harvest solar energy during the day, particularly in outdoor settings where electricity is often unavailable, and store it in the internal battery textiles. The stored energy can then be used to charge electronic devices like phones, smartwatches and earphones, especially at night when solar power is not available (Fig. 5g). We demonstrated that the charged textile batteries were effectively able to power a smartphone (Fig. 5g).

## CONCLUSION

In summary, we have broken the intrinsic micro-dimension manufacturing limitations of Li metal to develop a micrometer-thin and mechanically strengthened Li metal wire and have demonstrated its applicability in precise prelithiation of FLIBs and high-energy-density FLMBs. These Li metal wires are produced by a scalable molten-infusion process, during which the molten metallic Li is loaded into commercially available lithiophilic Ag/AY matrices. Benefiting from the tunable void volumes within the bundled Ag/AYs, the fabricated ultrathin Ag/AY@Li wires exhibit controllable diameters (20 to 534 μm) and ultralow capacities (0.0048 to 2.4 mAh cm^−1^). These diameters and capacities are one to three orders of magnitude lower than those of existing Li metal rods. Benefiting from their controllable and ultralow capacities, these Ag/AY@Li wires appropriately compensate for the loss of capacity during the initial cycle of fiber graphite anodes. As a result, the ICEs of the anodes are increased from 88% to 100%, achieving precise prelithiation. In addition, when employed as anodes, these ultrathin Ag/AY@Li wires also achieve outstanding stripping/plating stability because of the 3D host structure of the Ag/AY scaffolds and the derived Ag layer, resulting in a long lifecycle of 150 cycles with a capacity retention of 70.3% for the FLMBs, even under harsh conditions with a relatively low N/P ratio of 2.06. Moreover, the FLMB also delivers a high energy density of 139.822 Wh kg^−1^.

## METHODS

Details of the materials and methods are available in the online supplementary file.

## Supplementary Material

nwae480_Supplemental_File
