# Supplementary material for: Ultrathin and capacity-tunable lithium metal wires for lithium-based fiber batteries
Source: Natl Sci Rev. 2024 Dec 31;12(3):nwae480. doi: 10.1093/nsr/nwae480 (PMC11809254; doi:10.1093/nsr/nwae480)
Supplement: nwae480_Supplemental_File [file nwae480_supplemental_file.pdf]

Supporting Information

Ultrathin and capacity-tunable lithium metal wires for lithium-based fiber batteries

Chuanfa Li,† Qian Ye,† Jiaqi Wang, Xinlin Huang, Tianbing Song, Kun Zhang, Pengzhou Li, Yanan Zhang, Xiaocheng Gong, Yi Jiang, Yue Gao, Huisheng Peng and Bingjie Wang*

State Key Laboratory of Molecular Engineering of Polymers, Department of Macromolecular Science, Institute of Fiber Materials and Devices, and Laboratory of Advanced Materials, Fudan University, Shanghai 200438, China.

***Corresponding author**. E-mail: wangbingjie@fudan.edu.cn

†Equally contributed to this work.

This file includes:

Materials and Methods (Pages S2-S4)

Supplementary Figures 1 to 34 (Pages S5-S38)

Supplementary Table 1 (Page S39)

Supplementary Note 1 (Page S40)

Supplementary References (Page S41)

Materials and Methods

Fabrication of Ag/AY@Li wires. Ag/AY (WNK-130, WNK-300+, and WNK-500+) with various numbers of filaments (*i.e.*, 63, 133 and 200) were purchased from Wiyi New Materials Co., Ltd. The Ag/AY@Li wires were prepared by a fast capillary infusion of molten Li into the oriented channels in bundled Ag/AYs at a temperature of 300 °C. This infusing process was performed in an Ar-filled glove box with water and oxygen content <0.1 ppm. The loading of Li was achieved by simply immersing the Ag/AY yarn into the molten Li. To prepare the Ag/AY@Li-1, 2, 4, and 8 wires with lower capacities and diameters, Ag/AY-1, 2, 4 and 8 were made by drawing different numbers of filaments (1, 2, 4 and 8, respectively) from silver-plated poly-p-phenylene benzobisoxazole yarns as scaffolds. The fabrication process was similar to that of Ag/AY@Li-60, 130 and 200 wires but with a lower infusing temperature of 200℃.

Fabrication of fiber electrodes. To produce the fiber cathodes, two layered oxides, $LiCoO_2$ (LCO) and $LiNi_{0.8}Co_{0.1}Mn_{0.1}O_2$ (NCM811), were used as cathode materials. LCO fiber cathodes were fabricated as described previously[1]. Typically, polyvinylidene fluoride (PVDF), Super-P (SP) and LCO in weight ratios of 5:3:92 were sequentially added to N-methyl-2-pyrrolidone and evenly mixed in a planetary centrifugal mixer. The solid content of the slurry was 54 wt%. Aluminium wire with a diameter of 200 μm was selected as the current collector and dipped in the slurry and continuously drawn out to load the slurry, followed by passing through a furnace with a temperature of 120□ to dry the coating layer. Subsequently, the resulting fiber was further dried in vacuum at 80□ for 24 h. A similar procedure was followed to produce NCM811 fiber cathodes, while the weight ratios of PVDF, SP and NCM811 were 4:4:92 and the solid content of the slurry was 61 wt%.

Graphite fiber anodes were also fabricated as described previously[1]. The graphite slurry was formulated by mixing graphite, SP, sodium carboxymethyl cellulose, and butadiene styrene rubber at weight ratios of 93:2:1.4:3.6 in water, and stirring in a planetary centrifugal mixer. The solid content for the negative slurry was 50%. The electrode coating process mirrored that of the positive electrodes, with copper wires used as the current collector and a furnace drying temperature of 110□. Finally, the resulting graphite fiber anode was further dried in vacuum at 60□ for 24 h.

Prelithiation of fiber graphite anode. The fiber graphite anode was fed into a winding device, where it was wrapped with Ag/AY@Li wires (Ag/AY@Li-2, Ag/AY@Li-4, and Ag/AY@Li-8) as it moved through the system. Two motors controlled the collection speed and tension of the wrapping process, ensuring a consistent and uniform wrapping angle around the anode (**Fig.**

S34). This method enabled an even and efficient wrapping of Li wire onto the fiber anodes, enhancing system's scalability for potential industrial applications.

The fiber graphite anode was wrapped with an Ag/AY@Li wire, with a length two (for the Ag/AY@Li-8) or three (for the Ag/AY@Li-2 and Ag/AY@Li-4) times longer than the negative fiber. The wrapped negative fiber or bare negative fiber, each with a length of 1 cm, were assembled in CR2032 coin cells, with Li foils as reference and counter electrodes. 30 μL electrolyte containing 1 M $LiPF_6$ in ethylene carbonate–diethyl carbonate–ethyl methyl carbonate (KLD-1230C, Guangdong Canrd New Energy Technology) was added. Celgard 2325 separators were used to separate the wrapped negative fiber and Li foil. The OCV, ICE and cycling data of the half cells were collected using LAND battery test systems. The half cells were galvanostatically cycled at a rate of 0.3 C (1 C = 340 mA·g^{-1}) with an operating voltage window of 0.005−1 V after the 1st cycle at 0.1 C.

Assembly and test of FLMB. The positive fibers were wrapped with 3-mm-width separator strips (Celgard 2325). Then, Ag/AY@Li wire anodes were twisted with positive fibers. Fluorinated ethylene propylene tubes with a thickness of 0.5 mm were employed as the package of FLMBs. A dual-salt electrolyte containing 1.5 M lithium oxalyldifluoro borate and 1.0 M lithium tetrafluoroborate in a mixture of fluoroethylene carbonate and diethyl carbonate (in a ratio of 1:2, v:v) was injected into the encapsulation tube by an injector in a glove box. The FLMB was rested in the glove box for 12 h before use. The FLMB was galvanostatically charged at 0.3 C rate from 3 to 4.4 V and then charged at 4.4 V until current below 0.05 C rate, followed by galvanostatically discharging to 3 V at 0.5 C.

Fabrication of the integrated textile. The FLMBs woven in textiles were assembled with the similar fabrication process as above described, with the addition of separators wrapped around Ag/AY@Li wire anodes. The fiber solar cells produced following methods outlined in our earlier work[2]. Both the FLMBs and fiber solar cells were then woven on a rapier loom to create a large-area integrated textile.

Characterizations. The morphology and microstructure of AY, Ag/AY, Ag/AY-Li and fiber electrodes were observed by field emission scanning electron microscopy (FESEM, Ultra55). Fourier transform infrared (FTIR) spectra of AYs were performed by IR spectroscopy (Thermofisher Nicolet 6700) with the KBr disk method. Thermogravimetric analysis (TGA) was conducted using a Mettler Toledo TGA1 thermogravimetric analyzer under an oxygen atmosphere, with a heating rate of 10 □·min^{-1}, ranging from 30 to 1000□ in the N_2 atmosphere. The stress-strain curves of Ag/AY-Li and Li rod were obtained using a universal testing machine

(Instron 5565A tester) with a tensile speed of 10 mm·min^{-1}.

Electrochemical measurements. The electrolyte used for the symmetric fiber cells consisted of 30 μL of 1.0 M lithium bis(trifluoromethanesulfonyl)imide in a mixture of 1,3-dioxolane and 1,2-dimethoxyethane (1:1, v/v) with 1 wt% $LiNO_3$ as additive. Celgard 2325 was used as the separator. Symmetric fiber cells were cycled under various current density conditions to survey the Li plating/stripping behavior. Electrochemical impedance spectra of symmetric cells were measured by applying an AC amplitude of 5 mV over a frequency range from 0.01 to 10^5 Hz. To demonstrate the electrochemical stability of AY, a CV test was performed at a potential range of −0.5 to 5.0 V, with a scan rate of 1 mV·s^{-1}, in a CR2023 coin cell in which AY acted as the working electrode and Li foil served as the counter and reference electrode. Both CV and EIS tests were conducted using an electrochemical station (CHI660E, Chenhua).

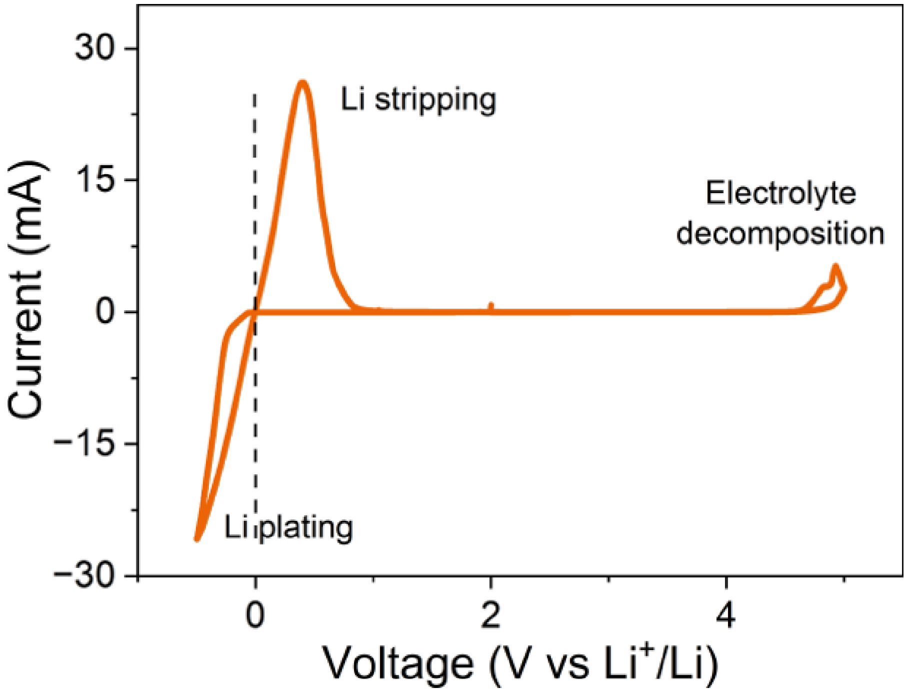

Fig. S1. CV curves of AY.

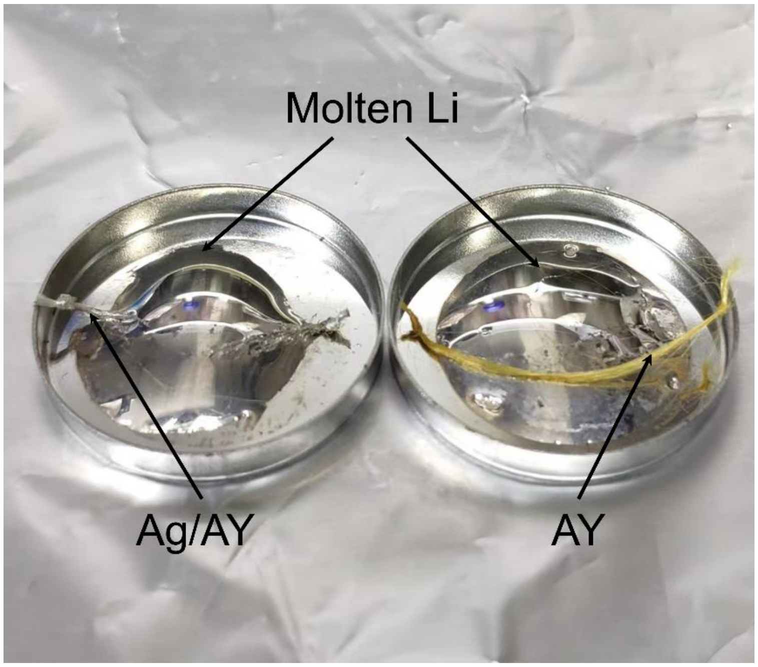

Fig. S2. Photographs of Ag/AY and AY immerged in molten Li.

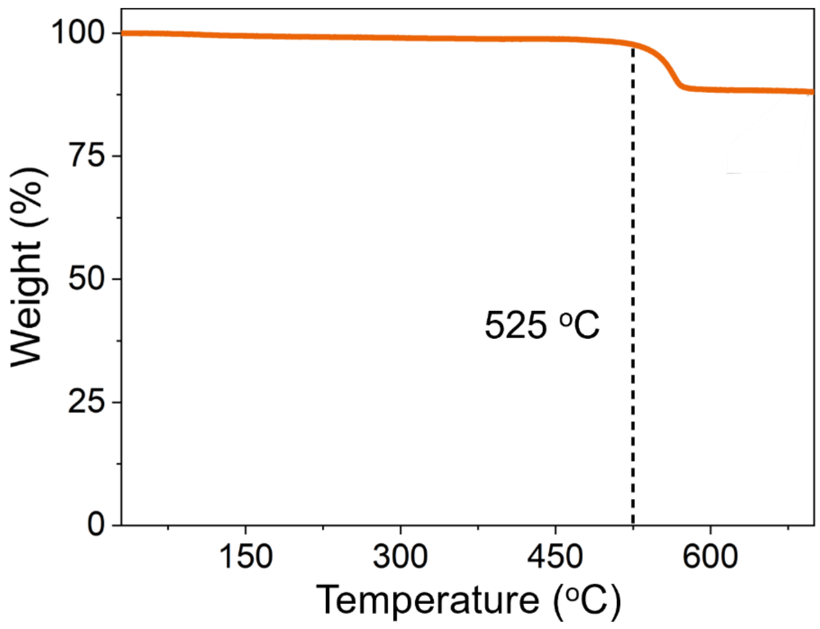

Fig. S3. TGA curve of Ag/AY.

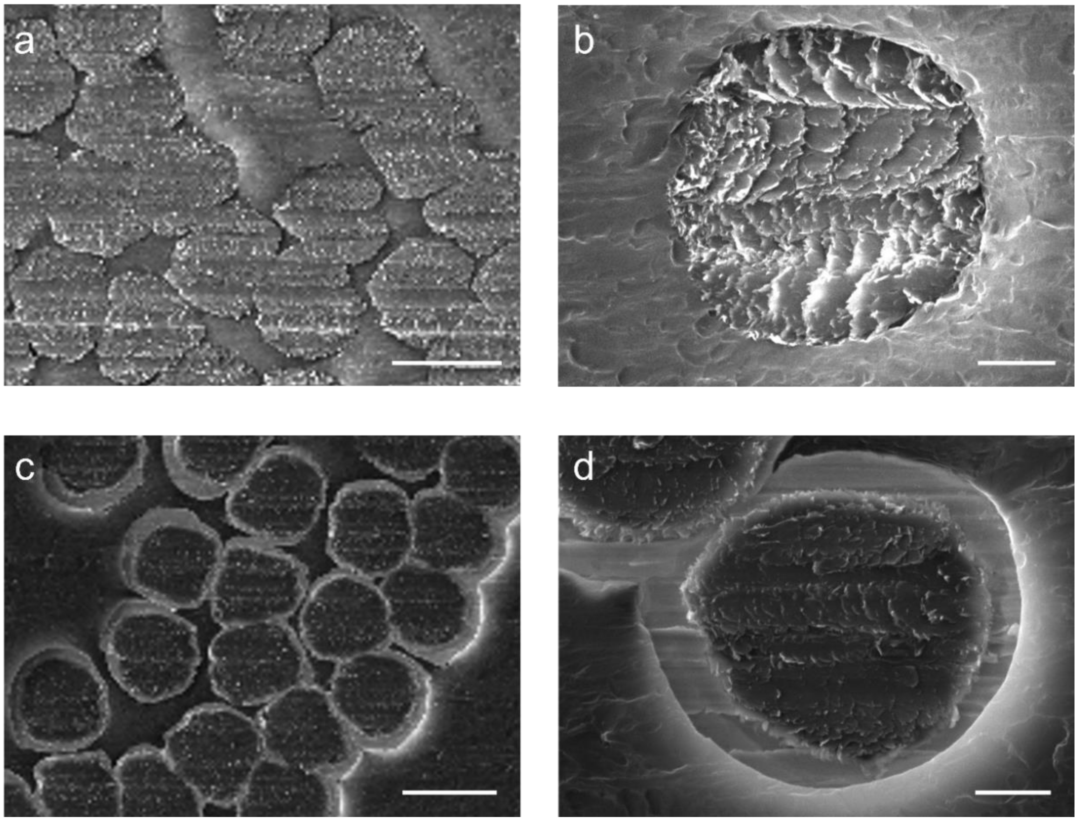

Fig. S4. Cross-sectional SEM images of AY (**a, b**) and Ag/AY (**c, d**). Scale bars: (a, c) 20 μm; (b, d) 5 μm.

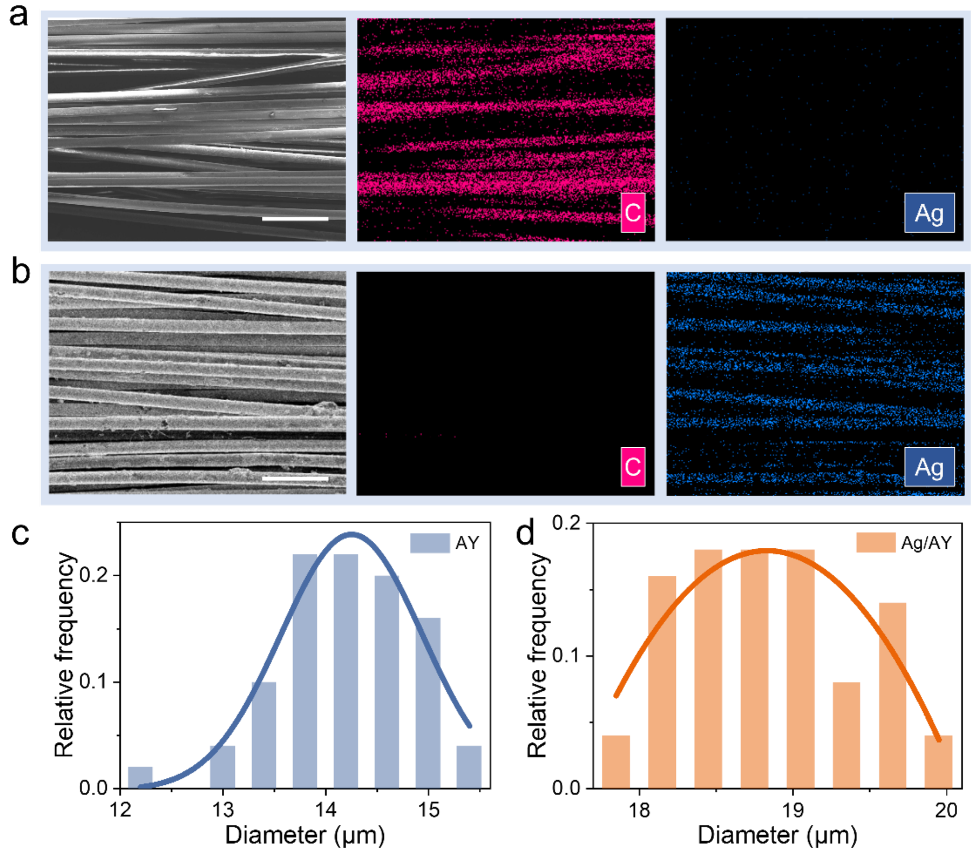

Fig. S5. a, b SEM images and corresponding C and Ag elemental mappings of AY (**a**) and Ag/AY (**b**). **c, d** Diameter distribution histograms of AY (**c**) and Ag/AY (**d**) from the SEM images. Scale bar: (a, b) 100 μm.

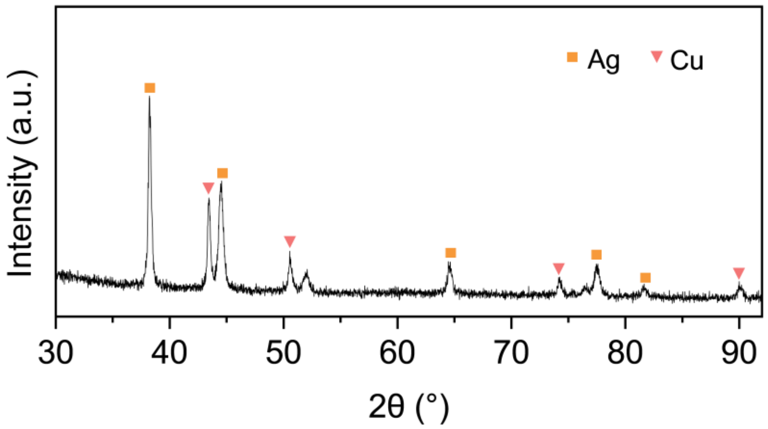

Fig. S6. X-Ray diffraction pattern of Ag/AY.

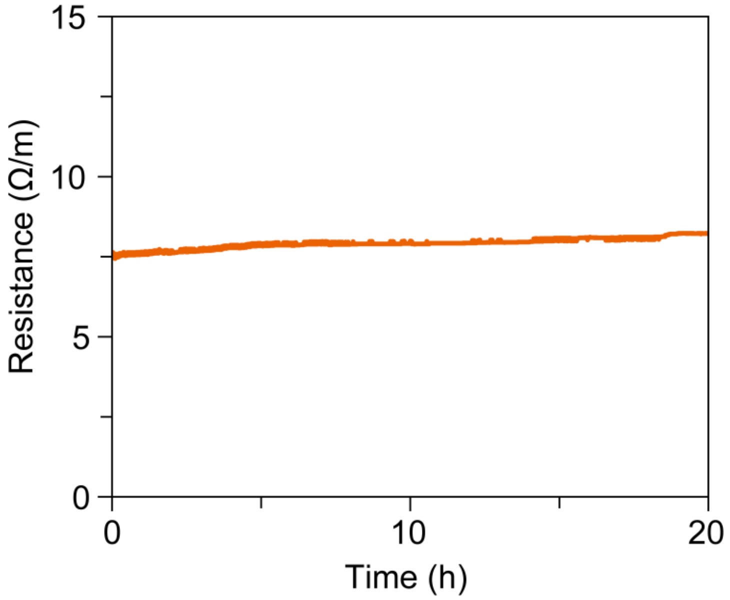

Fig. S7. Resistance change of the Ag/AY over time.

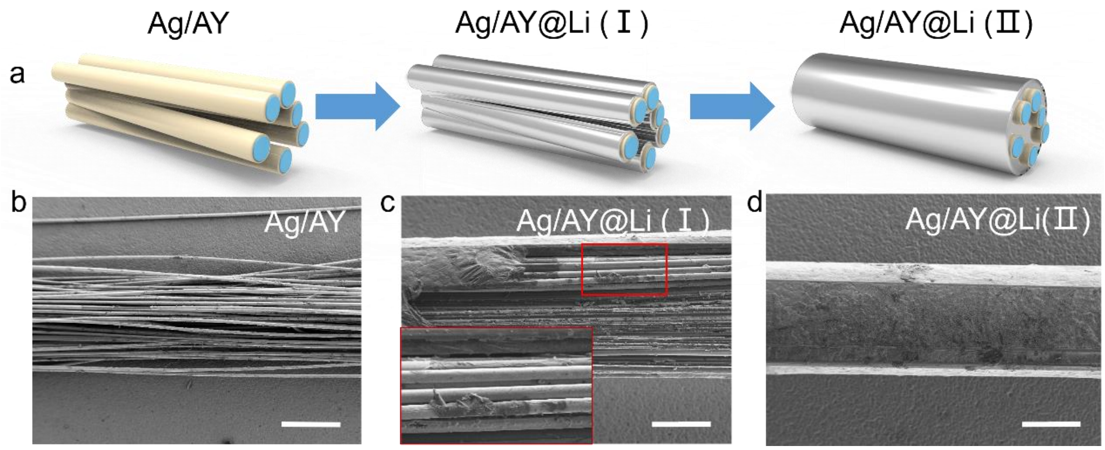

Fig. S8. Morphological change of Ag/AY during the infusion process. Scale bars: (b) 400 μm; (c, d) 200 μm.

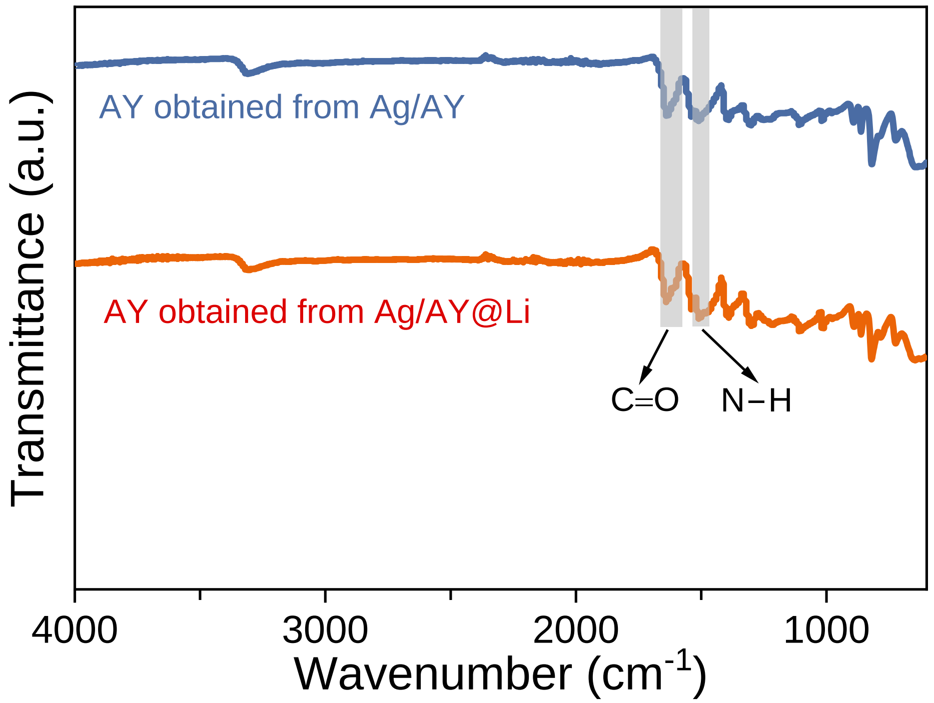

Fig. S9. FTIR spectra of the AY obtained from Ag/AY and Ag/AY@Li.

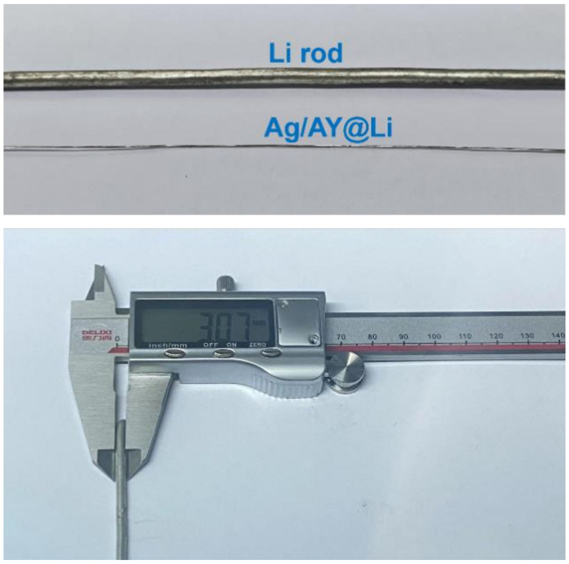

Fig. S10. Photographs of commercially available Li metal rod and our Li wire.

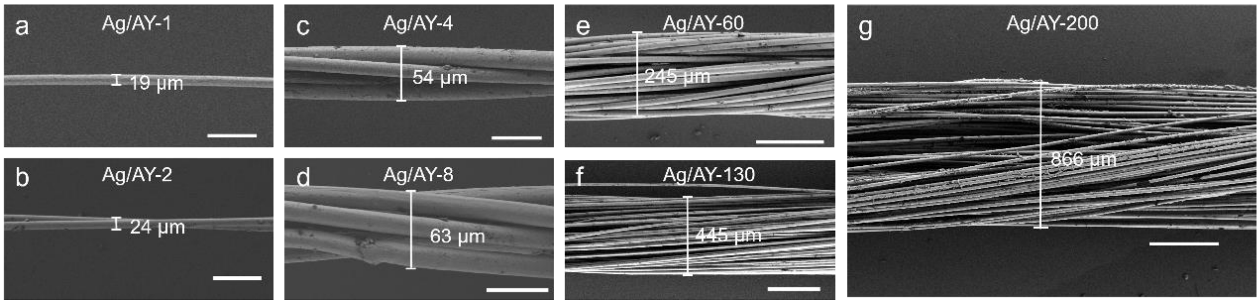

Fig. S11. SEM images of Ag/AYs with various numbers (1, 2, 4, 8, 60, 130 and 200) of the filaments. Scale bars: (a, b) 100 μm; (c, d) 50 μm; (e) 200 μm; (f) 300 μm; (g) 400 μm.

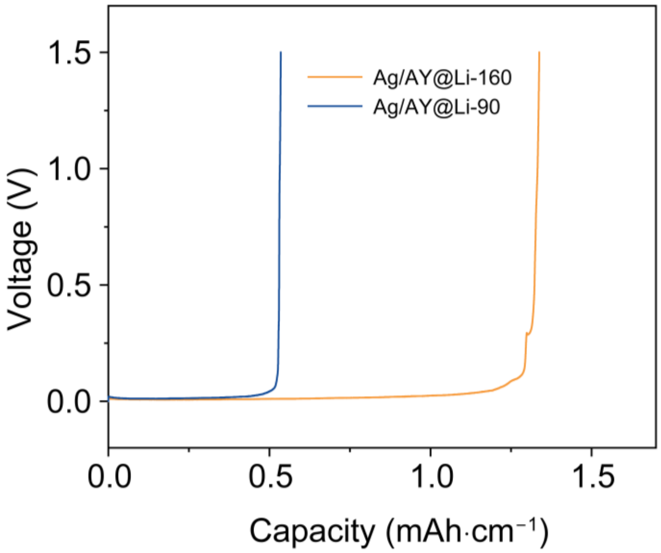

Fig. S12. Full Li stripping curves of Ag/AY@Li-90 and Ag/AY@Li-160.

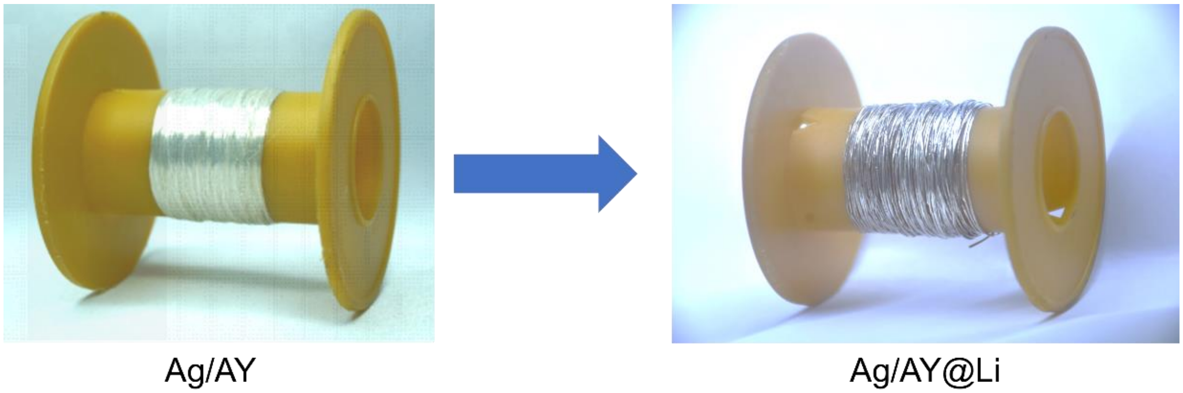

Fig. S13. A coil of Ag/AY (left) and Ag/AY@Li (right).

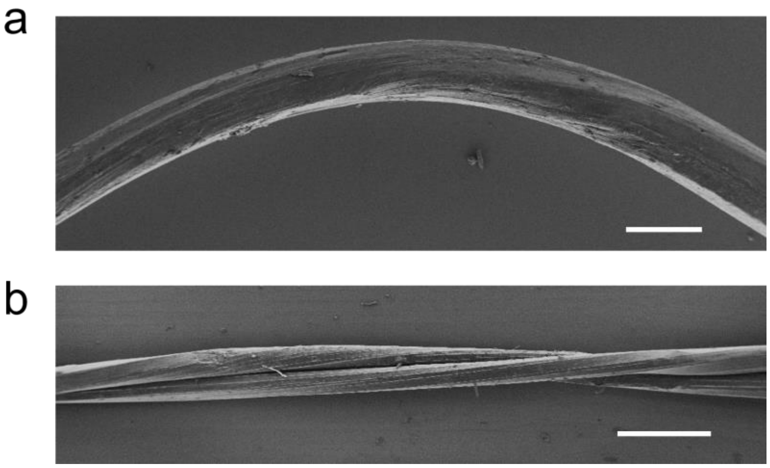

Fig. S14. SEM images of Ag/AY@Li wires after bending (**a**) and twisting (**b**), showing high flexibility of the obtained Li wire. Scale bar: (a) 300 μm, (b) 1 mm.

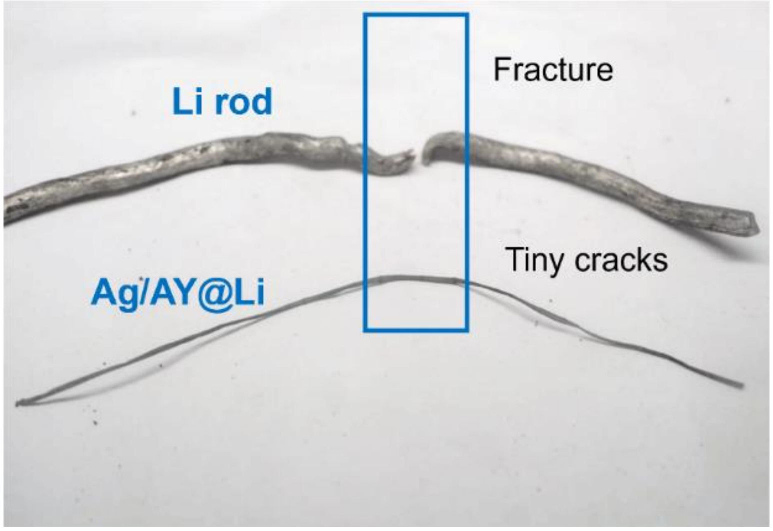

Fig. S15. Li rod Ag/AY@Li after repeatedly bending for 50 cycles.

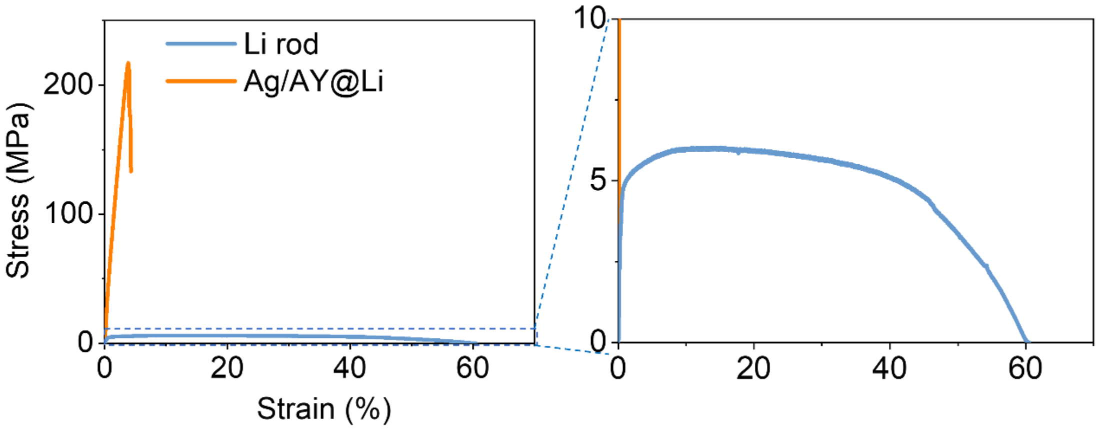

Fig. S16. Tensile stress-strain curves of the Ag/AY@Li wire and commercially available Li rod.

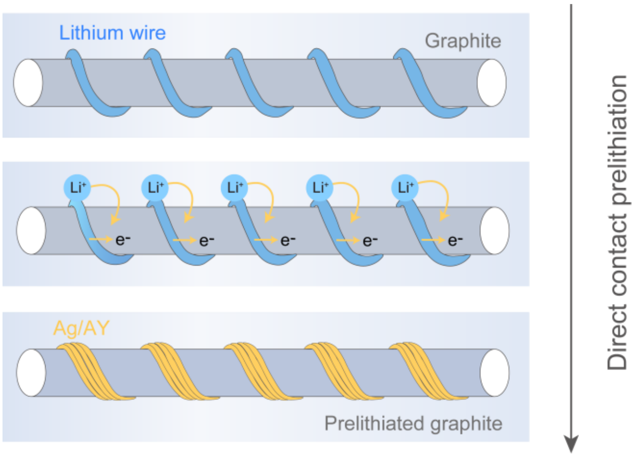

Fig. S17. Schematic diagram of the mechanism of direct contact prelithiation.

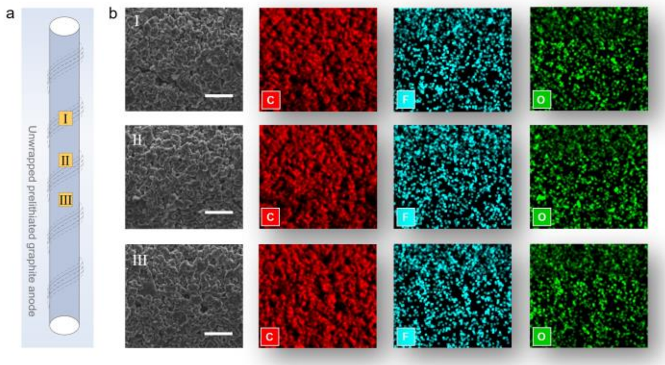

Fig. S18. (**a**) Schematic representation of the unwrapped prelithiated graphite anode highlighting the three testing positions. (**b**) SEM images and elemental mappings of the unwrapped prelithiated graphite anode at the three designated testing positions.

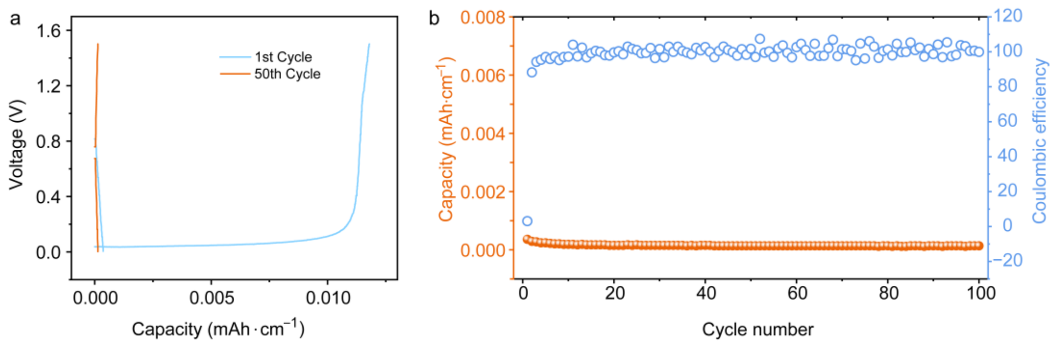

Fig. S19. (a) Galvanostatic charge and discharge curves of the Ag/AY@Li wire. (b) Galvanostatic cycling of the Ag/AY@Li wire. The counter-electrode used in the above tests is Li-metal foil.

The Ag/AY@Li wire exhibits an initial Li stripping capacity of approximately 0.012 $mAh{\cdot}cm^{-1}$ while a relatively low reversible capacity of less than 0.00036 $mAh{\cdot}cm^{-1}$ (**Fig. S19a** and **b**). Given such a low cycling capacity, the impact of the Ag/AY wire on cycling performance remains negligible over extended cycles.

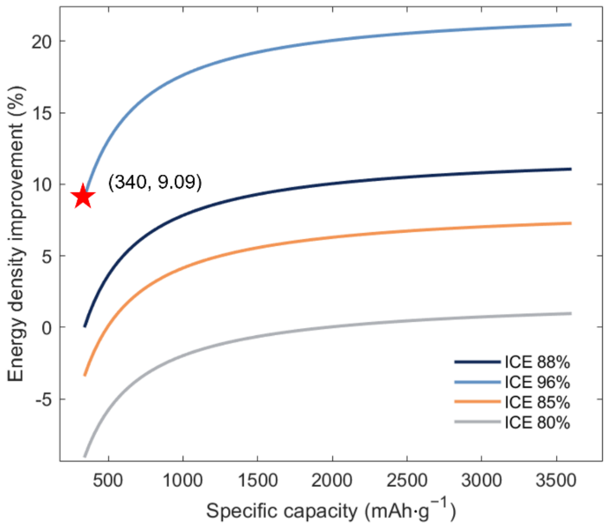

Fig. S20. Relationship between specific capacity and energy density enhancement of FLIB under different ICEs based on 11% of anode mass ratio and 88% of ICE.

In an LCO||Gr fiber battery system, the anode mass ratio is approximately 11%, with an ICE of about 88%. The mass ratio of Ag/AY-4 is approximately 0.8‰ of that of the FLIB, making it negligible in the calculation of energy density. Given a specific capacity of 340 mAh/g and an ICE of 88% for the graphite anode as the baseline, we can employ the following equation to calculate the relationship between the increase in energy density (y), the anode specific capacity (x), and the ICE.

For ICE of 88%, y = 100/(340*11/x+89)-1
For ICE of 96%, y = 100/ (340*11+89) *96%/88%-1
For ICE of 85%, y = 100/ (340*11+89) *85%/88%-1
For ICE of 80%, y = 100/ (340*11+89) *80%/88%-1

By using these equations, we can estimate the potential increase in energy density achieved through the precise prelithiation strategy.

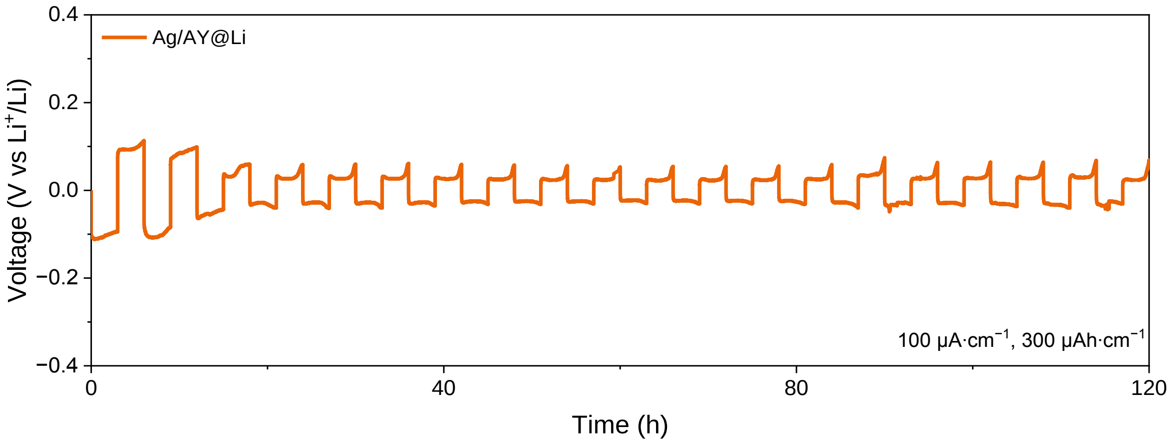

Fig. S21. Voltage-time profile of Li plating/stripping processes in fiber symmetric cell with Ag/AY@Li at 100 μA·cm^{-1} and 300 μAh·cm^{-1}.

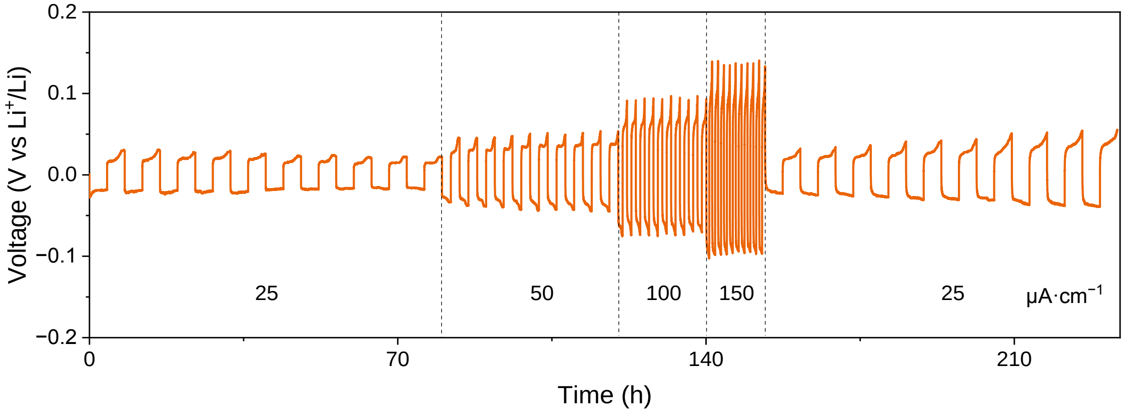

Fig. S22. Voltage-time profile of Li plating/stripping processes in fiber symmetric cell with Ag/AY@Li at various current densities with a fixed capacity of 100 μAh·cm^{-1}.

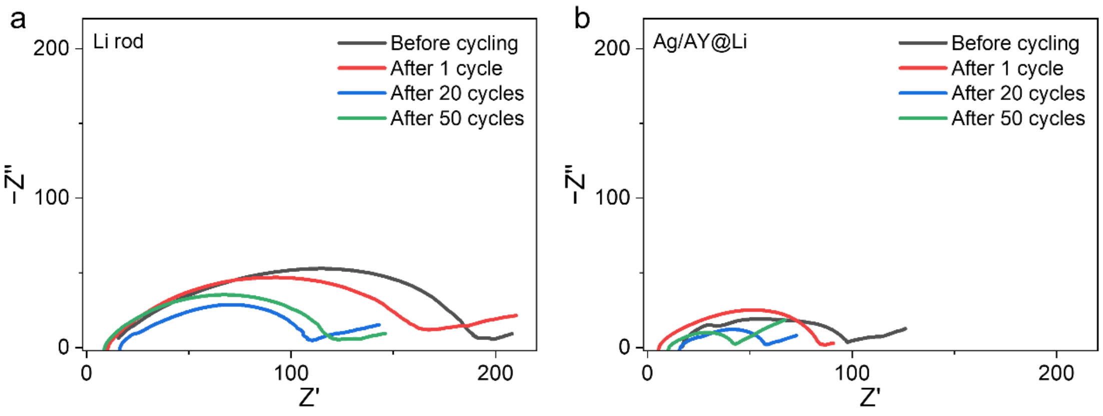

Fig. S23. Electrochemical impedance spectroscopies of the fiber symmetric cells with Li rod (**a**) and Ag/AY@Li (**b**) at different cycles at 50 μA·cm^{-1} and 50 μAh·cm^{-1}.

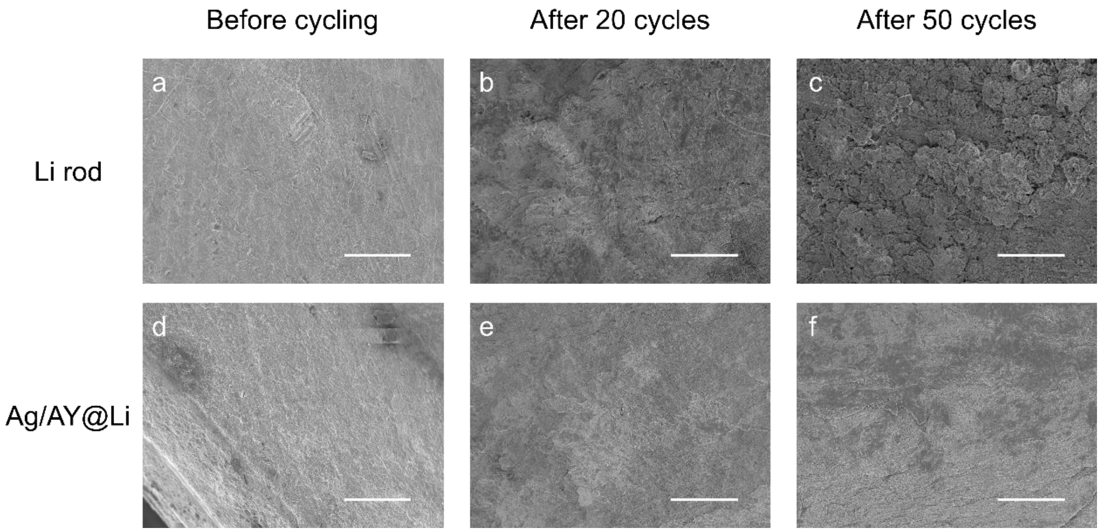

Fig. S24. SEM images of Li rods (**a–c**) and Ag/AY@Li wires (**d–f**) before cycling (**a, d**), after 20 (**b, e**) and 50 (**c, f**) cycles at 50 μA·cm^{-1} and 50 μAh·cm^{-1}. Scale bar: 50 μm.

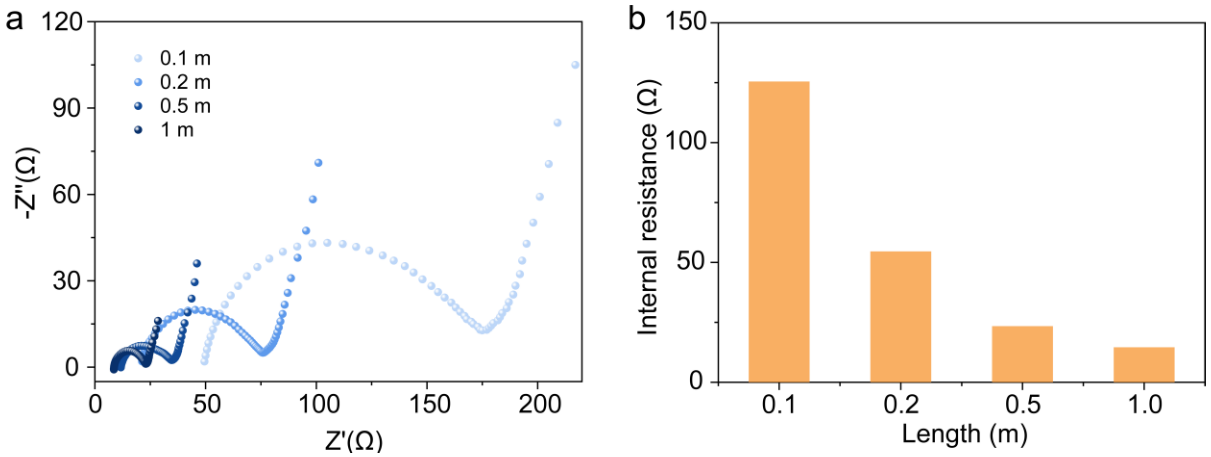

Fig. S25. (a) Electrochemical impedance spectroscopies of FLMBs with increasing lengths. (b) Relationship between internal resistance and length.

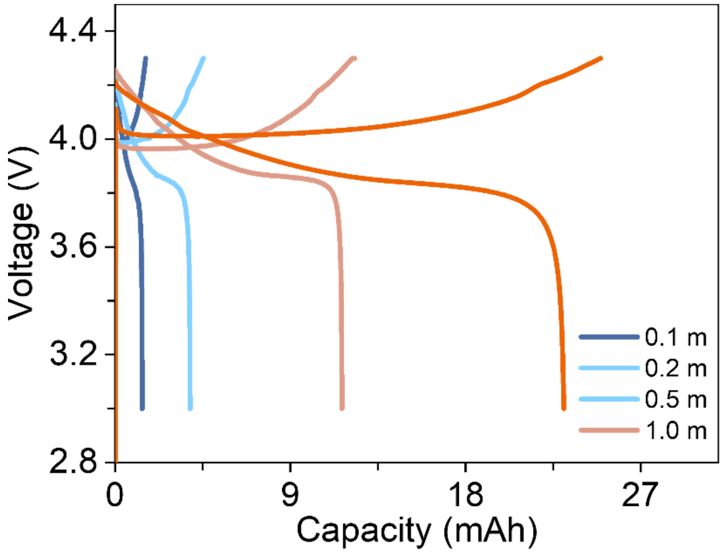

Fig. S26. Galvanostatic charge-discharge profiles of FLMBs with increasing lengths.

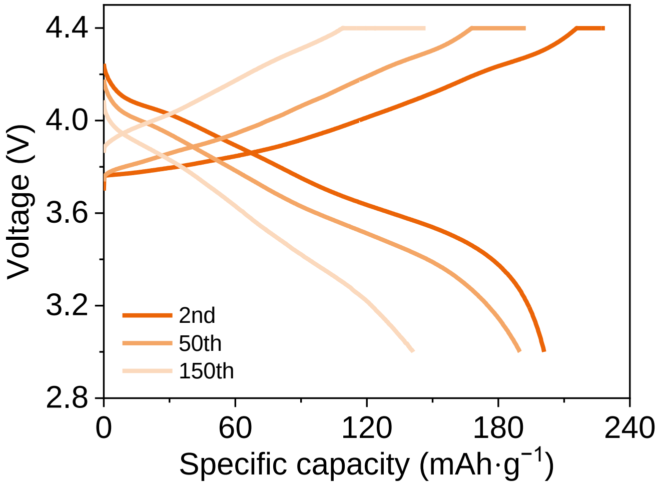

Fig. S27. Galvanostatic charge-discharge profiles of a 0.4-m-long FLMB.

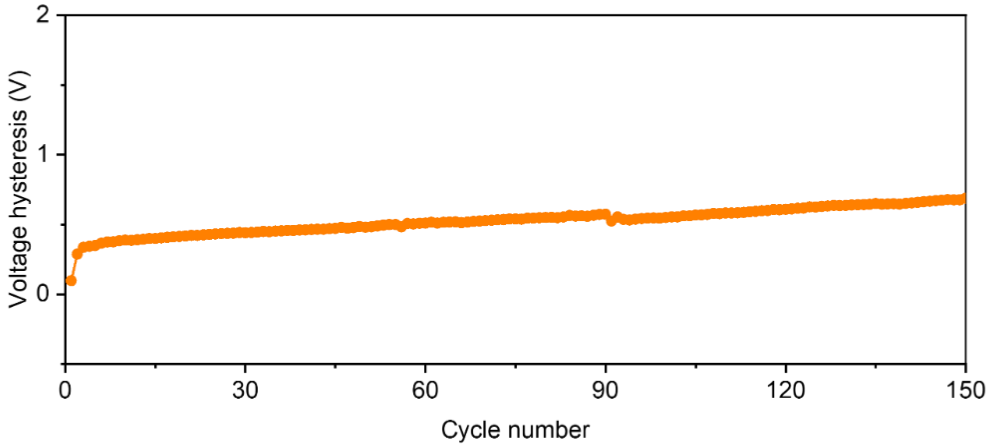

Fig. S28. Voltage hysteresis of a 0.4-m-long FLMB.

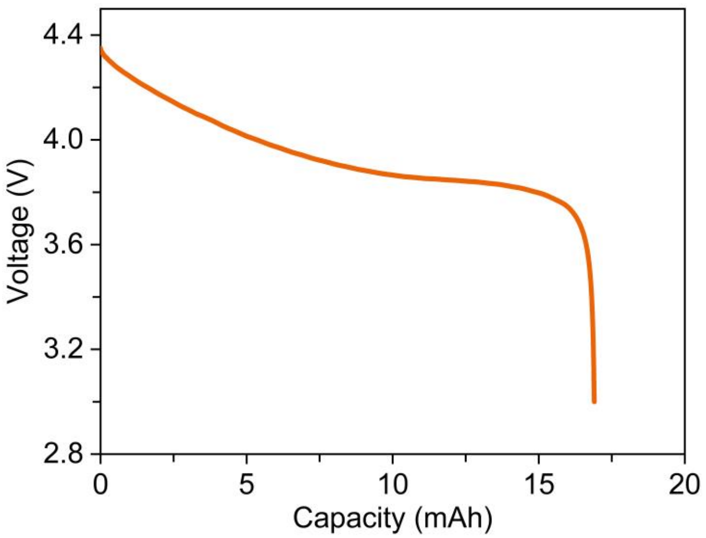

Fig. S29. Galvanostatic discharge profile of an FLMB.

For the capacity provided by the energy-storing textile, a 10 cm × 10 cm battery textile can accommodate approximately 20 single batteries, with each FLMB delivering a capacity of 16.901 mAh, resulting in a total capacity of about 338 mAh.

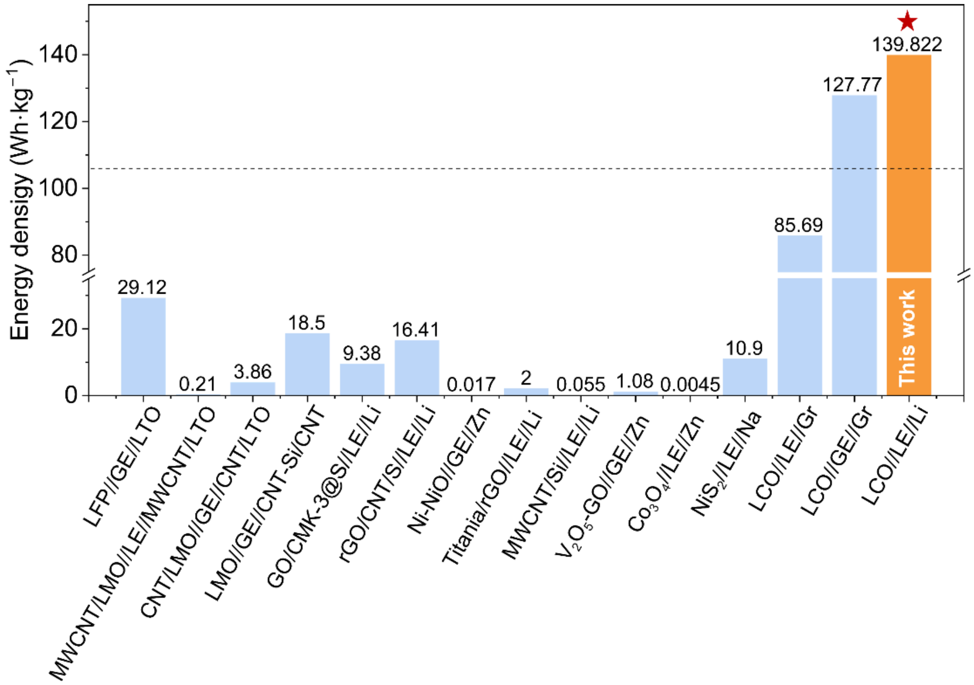

Fig. S30. Energy density of FLMBs compared with previously reported fiber batteries. The energy densities were calculated based on the total weight of the fiber batteries[1, 3-15].

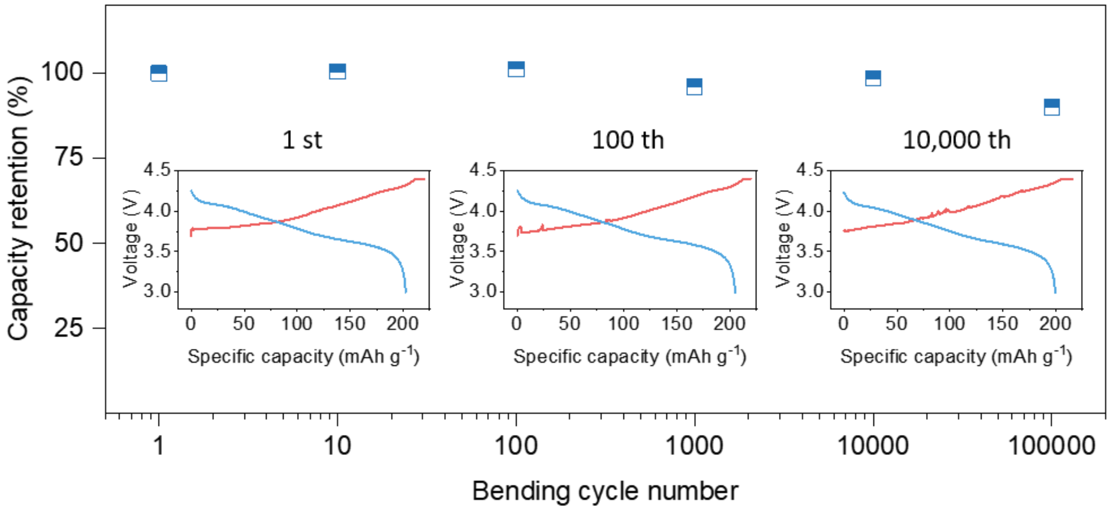

Fig. S31. Capacity retention under cyclic bending shows that 90% capacity is retained after 100,000 bending cycles.

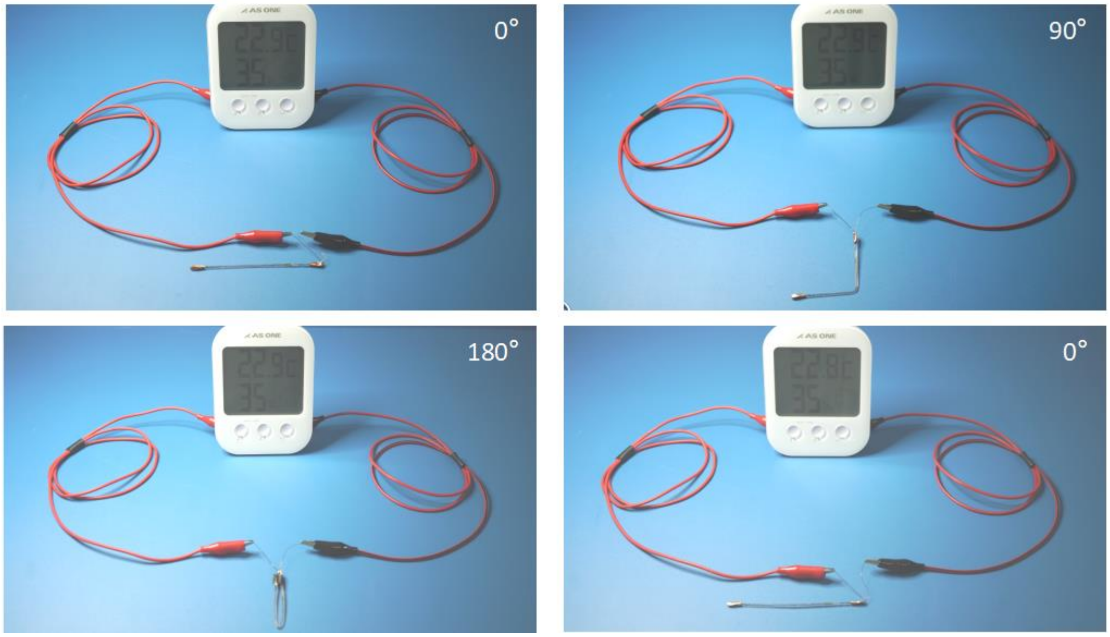

Fig. S32. An FLMB powers a hygrothermograph under different bending angles.

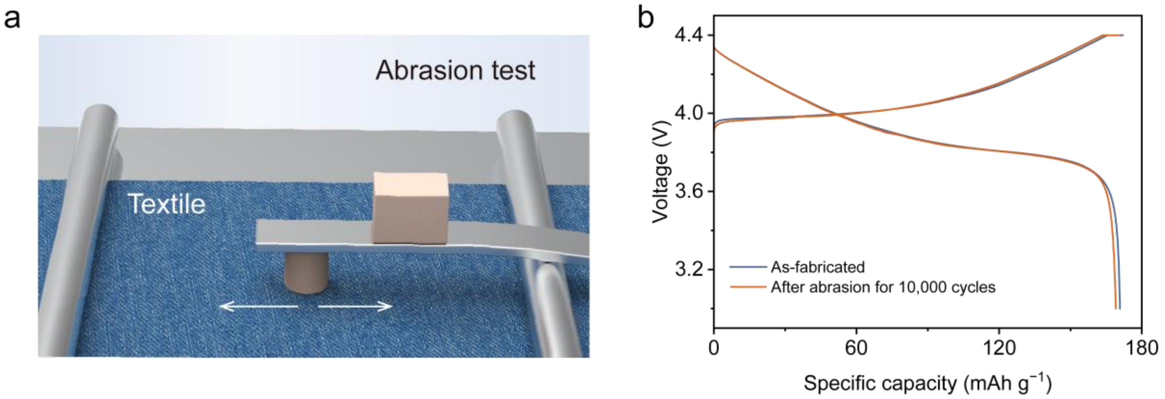

Fig. S33. Abrasion resistances of an FLMB textile. (**a**) Schematics of an FLMB textile under abrasion test. (**b**) Charge-discharge profiles of an FLMB textile before and after consecutive mechanical abrasion for 10,000 cycles.

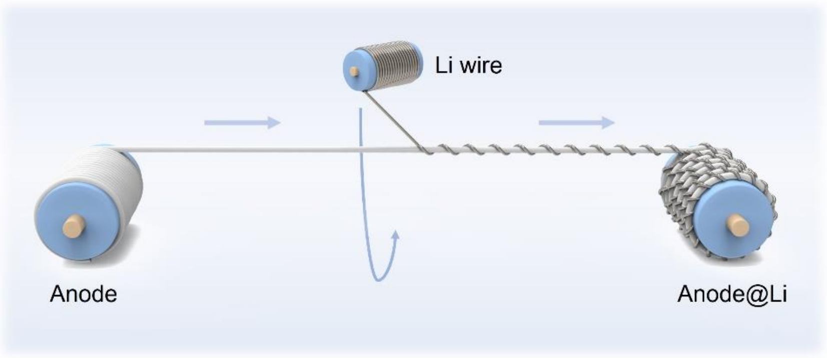

Fig. S34. Schematic illustrating wrapping Li wires around fiber graphite anodes continuously.

Table S1. The specifications of the FLMBs.

Component	Parameters
Cathode (mg)	131.075
Anode (mg)	7.28
Al current collector (mg)	59.2435
Ag/AF (mg)	27.14
Separator (mg)	9.5
Electrolyte (mg)	111.9615
Package (mg)	94.02
Taps (mg)	36.3
Total weight (mg)	476.52
Discharge capacity (mAh)	16.901
Discharge energy (mWh)	66.628
Specific energy ($Wh{\cdot}kg^{-1}$)	139.822

Supplementary Note 1

The practical specific capacity of the fiber anode considering the total weight of the electrode (Q_e) can be calculated by the following equation:

$$Q_e = \frac{Q_a}{m_a + m_c}$$

Where, Q_a is the linear capacity of the fiber anode, m_a represents the linear mass of anode materials and m_c stands for the linear mass of current collectors or scaffold.

$$Q_{\text{Graphite}} = \frac{100\,mg/m \times 340\,mAh/g}{100\,mg/m\ +\ 141\,mg/m} = 141.16\,mAh/g$$

$$Q_{\text{Ag/AY@Li-60}} = \frac{27\,mAh/m}{\frac{27\,mAh/m}{3860\,mAh/g} + 0.06\,g/m} = 403.02\ mAh/g$$

$$Q_{\text{Ag/AY@Li-130}} = \frac{82.2\,mAh/m}{\frac{82.2\,mAh/m}{3860\,mAh/g} + 0.16\,g/m} = 453.40\ mAh/g$$

$$Q_{\text{Ag/AY@Li-200}} = \frac{240\,mAh/m}{\frac{240\,mAh/m}{3860\,mAh/g} + 0.23\,g/m} = 821.42\ mAh/g$$

The above calculations show that the specific capacities of the Ag/AY@Li anodes are significantly higher than those of established graphite anodes. The enhanced specific capacity contributes to a substantial increase in energy density, highlighting the advantages of fiber Li anodes for applications such as smart textiles.

References

1. He J, Lu C, Jiang H *et al.* Scalable production of high-performing woven lithium-ion fibre batteries. *Nature* 2021; **597**: 57–63.
2. Zhu Z, Lin Z, Gu Y *et al.* Designing reflective hybrid counter electrode for fiber dye-sensitized solar cell with record efficiency. *Adv Funct Mater* 2023; **33**: 2306742.
3. Wang Y, Chen C, Xie H *et al.* 3D-printed all-fiber Li-ion battery toward wearable energy storage. *Adv Funct Mater* 2017; **27**: 1703140.
4. Ren J, Zhang Y, Bai W *et al.* Elastic and wearable wire-shaped lithium-ion battery with high electrochemical performance. *Angew Chem Int Ed* 2014; **53**: 7864–9.
5. Chen Q, Sun S, Zhai T *et al.* Yolk–shell NiS_2 nanoparticle-embedded carbon fibers for flexible fiber-shaped sodium battery. *Adv Energy Mater* 2018; **8**: 1800054.
6. Guan Q, Li Y, Bi X *et al.* Dendrite-free flexible fiber-shaped Zn battery with long cycle life in water and air. *Adv Energy Mater* 2019; **9**: 1901434.
7. Chong WG, Huang JQ, Xu ZL *et al.* Lithium–sulfur battery cable made from ultralight, flexible graphene/carbon nanotube/sulfur composite fibers. *Adv Funct Mater* 2017; **27**: 1604815.
8. Lin H, Weng W, Ren J *et al.* Twisted aligned carbon nanotube/silicon composite fiber anode for flexible wire-shaped lithium-ion battery. *Adv Mater* 2014; **26**: 1217–22.
9. Xia Z, Li S, Wu G *et al.* Manipulating hierarchical orientation of wet-spun hybrid fibers via rheological engineering for Zn-ion fiber batteries. *Adv Mater* 2022; **34**: 2203905.
10. Zeng Y, Meng Y, Lai Z *et al.* An ultrastable and high-performance flexible fiber-shaped Ni–Zn battery based on a Ni–NiO heterostructured nanosheet cathode. *Adv Mater* 2017; **29**: 1702698.
11. Weng W, Sun Q, Zhang Y *et al.* Winding aligned carbon nanotube composite yarns into coaxial fiber full batteries with high performances. *Nano Lett* 2014; **14**: 3432–8.
12. Hoshide T, Zheng Y, Hou J *et al.* Flexible lithium-ion fiber battery by the regular stacking of two-dimensional titanium oxide nanosheets hybridized with reduced graphene oxide. *Nano Lett* 2017; **17**: 3543–9.
13. Fang X, Weng W, Ren J *et al.* A cable-shaped lithium sulfur battery. *Adv Mater* 2016; **28**: 491–6.
14. Lu C, Jiang H, Cheng X *et al.* High-performance fibre battery with polymer gel electrolyte. *Nature* 2024; **629**: 86–91.
15. Zhang Y, Bai W, Cheng X *et al.* Flexible and stretchable lithium-ion batteries and supercapacitors based on electrically conducting carbon nanotube fiber springs. *Angew Chem Int Ed* 2014; **53**: 14564–8.
